# Genome-wide transcriptome profiling revealed biological macromolecules respond to low temperature stress in *Brassica napus* L

**DOI:** 10.3389/fpls.2022.1050995

**Published:** 2022-11-14

**Authors:** Muhammad Azhar Hussain, Dan Luo, Liu Zeng, Xiaoyu Ding, Yong Cheng, Xiling Zou, Yan Lv, Guangyuan Lu

**Affiliations:** ^1^ Key Laboratory of Biology and Genetic Improvement of Oil Crops Research Institute, Oil Crops Research Institute, Chinese Academy of Agricultural Sciences (CAAS), Wuhan, China; ^2^ School of Biology and Food Engineering, Guangdong University of Petrochemical Technology, Maoming, China

**Keywords:** abiotic stress, low temperature stress, RNA sequencing, DEGs, transcription factors, photosynthesis, antioxidants

## Abstract

*Brassica napus* L. (*B. napus*) is a vital oilseed crop cultivated worldwide; low temperature (LT) is one of the major stress factors that limit its growth, development, distribution, and production. Even though processes have been developed to characterize LT-responsive genes, only limited studies have exploited the molecular response mechanisms in *B. napus*. Here the transcriptome data of an elite *B. napus* variety with LT adaptability was acquired and applied to investigate the gene expression profiles of *B. napus* in response to LT stress. The bioinformatics study revealed a total of 79,061 unigenes, of which 3,703 genes were differentially expressed genes (DEGs), with 2,129 upregulated and 1,574 downregulated. The Gene Ontology and Kyoto Encyclopedia of Genes and Genomes enrichment analysis pinpointed that the DEGs were enriched in LT-stress-responsive biological functions and metabolic pathways, which included sugar metabolism, antioxidant defense system, plant hormone signal transduction, and photosynthesis. Moreover, a group of LT-stress-responsive transcription factors with divergent expression patterns under LT was summarized. A combined protein interaction suggested that a complex interconnected regulatory network existed in all detected pathways. RNA-seq data was verified using real-time quantitative polymerase chain reaction analysis. Based on these findings, we presented a hypothesis model illustrating valuable information for understanding the LT response mechanisms in *B. napus*.

## Introduction

*Brassica napus* is extensively grown and distributed in the Yellow River basin in China (Ke et al., 2020). *B. napus* is the third most important oil crop in China (Li et al., 2021). To date, *B. napus* has become a research hotspot due to its commercial and ecological benefits (Liu et al., 2019). It has long been known that environmental concerns such as low temperature (LT) stress, including chilling (0–10°C) and freezing (<4°C), affect the germination, growth, development, production, and spatial distribution of crops. In recent years, winter- and semi-winter rapeseeds cultivated in the Yangtze River basin were sowed from the end of September to mid-October, which leads to threatened growth and production of rapeseed under LT stress conditions (Cong et al., 2019; Huang et al., 2020). Improvement in cold tolerance has been a major goal for the agricultural research of *B. napus* over the recent years; there is an urgent need to develop and cultivate early-maturing rapeseed varieties with cold resistance (Raza et al., 2021).

Over the years, available literature has reported LT-responsive genes and regulatory mechanisms in *Arabidopsis* (Du et al., 2017; Yu et al., 2020). The researchers focused on elucidating the LT response molecular regulatory mechanisms in different crop species such as tomato (Niu et al., 2022), apple (An et al., 2018), banana (Liu et al., 2018a), rice (Lv et al., 2017; Hang et al., 2018), rapeseed (Luo et al., 2019; Pu et al., 2019; Huang et al., 2020), and maize (Li et al., 2016b). These efforts revealed numerous genes, molecular regulators, and biological pathways to ameliorate plant LT stress effects (Shi et al., 2018; Ding et al., 2019). To cope with unfavorable environmental factors, increasing attention has been given to understanding the cold response mechanisms in oilseed crops, especially rapeseed—for example, next-generation sequencing (NGS) technology is widely applied to reveal the transcriptomic changes in rapeseed under different stresses (Xiong et al., 2022). Recently, the transcriptomic analysis identified various cold-responsive (COR) pathways that regulate photosynthesis, abscisic acid (ABA) homeostasis and transport, plant hormone signal transduction, ribosome biogenesis, MAPK signaling pathway, calcium signal transduction, and antioxidant defense systems in rapeseed (Ma et al., 2019). Different studies reported that the accumulation of different metabolites, including proline, soluble sugar, and protein contents, and antioxidant enzyme activity were rapidly increased under freezing stress (-2°C) in rapeseed (Yan et al., 2019). Dehydrins such as late embryogenesis abundant (LEA) proteins are accumulated in response to cold stress by a higher expression of *LEA10* and *LEA18*, *LEA90*, and *LEA104* genes in *B. napus* (Maryan et al., 2019). LT stress-inducible proteins are antifreeze proteins, cold-shock domain proteins, and heat shock proteins (HSPs). Under LT stress, various enzymes, such as oxidases and desaturases, are activated to scavenge the reactive oxygen species (Heidarvand and Maali, 2010; Su et al., 2021). Among different antioxidant enzymes, superoxide dismutase (SOD), peroxidase (POD), ascorbate peroxidase (APX), and catalase (CAT) act as the first line of reactive oxygen species (ROS) scavenging to protect stress-induced oxidative damage in plants (Sharma et al., 2019; Su et al., 2021).

The phenotypic and RNA-seq profiling of transgenic *B. napus* ectopic overexpressing *Arabidopsis* C-repeat/DRE-binding factor (CBF) has suggested that the CBF cold response of *Arabidopsis* has been maintained in rapeseed (Jaglo et al., 2001). In the grape plant, CBL-interacting protein kinases 18 act as a positive regulator of the CBF cold signaling pathway by modulating ROS homeostasis (Yu et al., 2022b). Plant-hormone-mediated pathways are also critical in plant LT stress tolerance—for example, sly-miR156e-3p mediated the posttranscriptional regulation of transcription factor S1MYB15, which positively regulates ABA-mediated cold tolerance in the tomato (Zhang et al., 2022b). In the last decade, different efforts have been made to investigate the physiological and molecular perspectives of LT response of *B. napus* (Du et al., 2016; Huang et al., 2018; Xin et al., 2019; Ke et al., 2020). Despite these efforts, the complex molecular mechanisms of LT stress responses in *B. napus* need to be further explored. The NGS profoundly helped to dissect genome-wide novel and diverse molecular response mechanisms in plants. These NGS technologies provide simple, low-cost, sensitive, accurate, and fast tools for elucidating the genome-wide regulation of desired traits (Tyczewska et al., 2016; Sharma et al., 2018). The RNA-seq technology has many successful stories in profiling the genetic architecture of LT stress tolerance, including *B. rapa* (Ma et al., 2019), *B. oleracea* (Zhang et al., 2019c), *B. napus* (Du et al., 2016; Ke et al., 2020), *A. thaliana* (Kaplan et al., 2007), *Z. mays* (Li et al., 2016b; Li et al., 2019), *O. sativa* (Guan et al., 2019), and *E. japonica* Lindl (Zhang et al., 2022a).

Based on the abovementioned details, the molecular picture associated with cold response has become clearer. *B. napus* was capable of growth at LT conditions; however, the cold responses of the early-maturing variety in a short time remain to be characterized. In this study, we assessed the dynamic changes that occur at the molecular level to elucidate the LT stress responses through biochemical and transcriptome analysis in *B. napus*. Biochemical analysis helps to identify the activation of different kinds of phytohormones under LT stress. The transcriptome analysis helps explore various COR gene pathways and their regulatory mechanisms operating under LT conditions in *B. napus*. The data generated in this study would enhance the understanding of LT response and regulatory mechanism and identify substantial genetic resources that are useful for modifications and enhancing LT tolerance in *B. napus*.

## Materials and methods

### Plant materials, growth conditions, and treatments

Rapeseed Cultivar-18, an excellent inbred winter cultivar bred by Oil Crops Research Institute, Wuhan, China, that can be successfully cultivated under LT conditions, was used in this study. The seeds were germinated on moist filter paper in petri plates under soft tube white lights at a temperature range of 25 ± 1°C, humidity of 60%, and 16-h light/8 h dark photoperiod in a growth chamber. One-week-old seedlings were transferred to plastic pots filled with a 1:1:1 mixture of peat vermiculite/moss/perlite in a growth room (16 h light/8 h dark photoperiod, with a constant temperature of 25 ±1°C for approximately 21 days. At a 4-leaves stage, healthy seedlings were selected and transferred to LT stress (4°C) conditions. Before LT treatment (0 d) and after LT conditions (1 d), leaves were sampled, immediately frozen in liquid nitrogen, and preserved at -80°C until further use. Seedlings were recovered under normal growth conditions for two days after LT stress treatment. Total RNA was extracted from a pooled sample of seedling leaves from each group.

### RNA preparation and deep sequencing

RNA preparation and transcriptome analysis were performed as previously described (Raza et al., 2021). Briefly, total RNA was extracted from the leaves of LT stressed and non-stressed samples using the TRIzol kit (Invitrogen, USA). RNA concentration and quality were quantified using NanoDrop 2000 (Thermo). The RNA sample was prepared from 1 μg RNA per sample for sequencing. cDNA libraries were produced using NEBNext UltraTM RNA Library Prep Kit for Illumina (NEB, USA), following the manufacturer’s instructions at the Biomarker company (Beijing, China), and then sequenced on an Illumina platform employing paired-end technology. Clean data was further subjected to Q20, Q30, GC-content and subsequent analysis. Hisat2 tools software was used to map clean reads with the reference genome sequence of *B. napus* (https://www.genoscope.cns.fr/brassicanapus) (Chalhoub et al., 2014). The sequence data is publicly accessible on the National Center for Biotechnology Information database (PRJNA596550).

### Differential expression analysis

Differential expression analysis of data under two conditions, LT stressed and non-stressed, was performed using the DEseq, which provided gene expression data based on the negative binomial distribution. Gene expression level was measured by the number of fragments per kilobase of exon in per million fragments mapped reads (Trapnell, 2010). Benjamini and Hochberg’s method was used to control the false discovery rate (Benjamini and Hochberg, 1995). Genes with an adjusted *P*-value <0.001 were considered differentially expressed genes (DEGs). Gene Ontology (GO) and Kyoto Encyclopedia of Genes and Genomes (KEGG) enrichment analyses were performed using a hypergeometric test with a false discovery rate adjusted *p*-value <0.05.

### Gene functional annotation

The functional annotations of all these obtained DEGs were explored using the BLASTx program to find out the NCBI non-redundant protein (NR) and *Arabidopsis* protein (TAIR10) databases. The DEGs were subjected to GO enrichment analysis using the GOseq R packages based on Wallenius non-central hyper-geometric distribution (Young et al., 2010). The Blast2GO program was used to categorize the GO terms into three major categories of molecular function, cellular component, and biological process (Conesa and Götz, 2008). The KEGG pathway annotation was performed using KOBAS software to elaborate the corresponding metabolic pathways (Mao et al., 2005).

### Physiological and biochemical analysis

To explore the physiological and biochemical changes under LT stress conditions, the contents of hydrogen peroxide (H_2_O_2_), malondialdehyde (MDA), soluble sugar, and proline (Pro) were quantified using commercial kits purchased from SolarBio (https://solarbio.com) following the manufacturer’s protocol. The antioxidant enzyme activities, including superoxide dismutase (EC 1.15.1.1), peroxidase (EC 1.11.1.7), catalase (EC 1.11.1.6), and ascorbate peroxidase (EC 1.11.1.11), were quantified following the SolarBio kits (https://solarbio.com) guidelines. The photosynthesis parameter Fv/Fm was measured using a portable chlorophyll-fluorometer OS-30p+ (OPTI-SCIENCES, China). All physiological and biochemical parameters were measured using a spectrophotometer microplate reader (Epoch, BioTek, Instruments, USA) in triplicate biological replicates.

### qRT-PCR validation

The RNA-seq data was validated through qRT-PCR analysis. The first-strand cDNA was reverse-transcribed from 1 μg DNA-free RNA using EasyScript^®^One-Step cDNA Synthesis SuperMix (Trans) according to the manufacturer’s instructions. Amplification was performed using a StepOnePlusReal-Time PCR System (Applied Biosystems) with a Power SYBR^®^Green PCR Master Mix according to the manufacturer’s instructions. The reaction protocol was as follows: 95°C for 10 m, 42 cycles at 95°C for 15 s, and 60°C for 15 s, with 10 ul as the final volume following three technical replicates. Relative expression data were analyzed using the 2^–ΔΔ^Ct algorithm using *B. napus* ACTIN as an internal control (Wang et al., 2014). The primers used for qRT-PCR are listed in **Supplementary Table S1**.

## Results

### Physiological and biochemical response to LT stress

**Figure 1:**
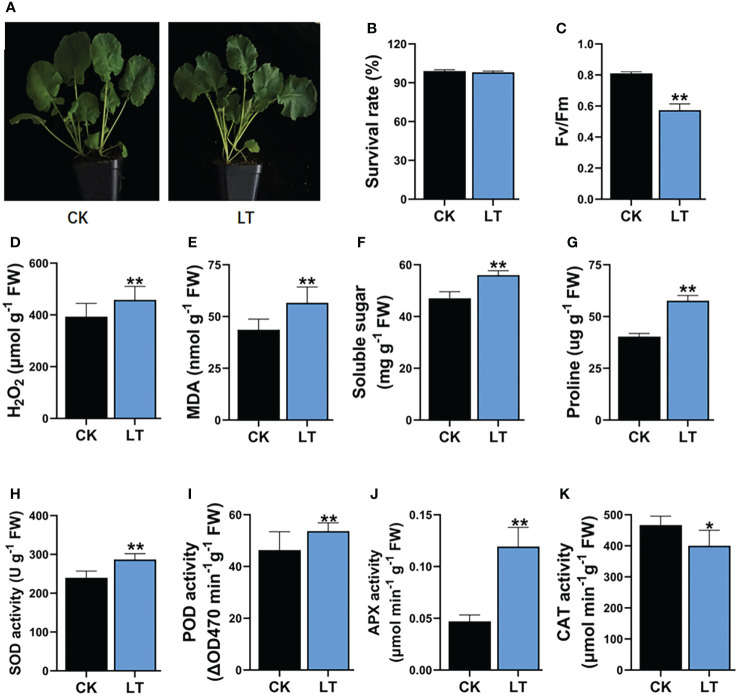
Impact of LT stress treatment on physiology and biochemical indexes in rapeseed. **(A)** Plant phenotype, **(B)** survival rate, **(C)** maximum quantum efficiency of photosystem II (PSII) Fv/Fm, **(D)** hydrogen peroxide (H2O2), **(E)** malondialdehyde (MDA), **(F)** soluble sugar, **(G)** proline contents, **(H)** superoxide dismutase (SOD) 1124 activity, **(I)** peroxidase (POD) activity, **(J)** ascorbate peroxidase (APX) activity, **(K)** 1125 Catalase (CAT) activity. Asterisks show significant levels at **P ≤ 0.01, *P ≤ 0.05.

LT stress has damaging effects, which impair the growth and productivity of *B. napus*. After a short-term 4°C stress treatment, *B. napus* seedlings showed >99% survival rate after 2 days of recovery (**Figures 1A, B**). However, 4°C-LT-stress-treated seedlings exhibited significant differences in physiological and biochemical indices compared with CK conditions. Therefore, the maximum quantum efficiency of photosystem II (PSII) Fv/Fm is reduced by 29.22% under LT stress treatment in rapeseed (**Figure 1C**). However, LT stress leads to the induction of H_2_O_2_ and MDA contents in plants. H_2_O_2_ plays a dual role in response to stress conditions and acts as a ROS and signaling molecule to activate the stress response mechanism in plants. However, excessive H_2_O_2_ accumulation is damaging to cells. LT stress treatment significantly increased the H_2_O_2_ and MDA levels—specifically, H_2_O_2_ increased by 16.52% and MDA increased by 29.77% (**Figures 1D, E**). Similarly, osmoprotectants, such as soluble sugars and proline, increased by 19.14% and 42.97% compared with CK, respectively (**Figures 1F, G**). These results indicate that the accumulation of osmoprotectants has a protective role in LT-stressed *B. napus* seedlings. In response to higher ROS, the plant defense system is activated to ameliorate the effects of LT stress compared with CK. The built-in plant defense system is comprised of various kinds of antioxidant enzymes. These antioxidant enzymes, including SOD, POD, CAT, and APX, equilibrate ROS production and are involved in the conversion to the least deleterious biochemicals within plant cells under LT stress. We observed that the LT stress significantly increased the SOD enzyme activity by 19.61%, POD enzyme activity by 15.82%, and APX enzyme activity by 19.69% compared with CK seedlings (**Figures 1H–J**). In contrast, CAT enzyme activity was reduced by 15.3% compared with CK (**Figure 1K**). These results indicate that, in *B. napus* seedlings, complex physiological and biochemical responses are simultaneously activated to protect them from the injurious effects of LT stress (**Figure 1**).

### RNA-seq and data quality control analysis

The current study used *B. napus* plants as experimental material to reveal transcriptome changes under LT stress. At the four-leaves stage, healthy plants were subjected to 4°C for 1 day under 16/8-h photo/dark period for LT treatment. The morphological changes in the leaves of *B. napus* plants were recorded. The phenotypic changes indicated that *B. napus* plants were challenged by LT stress (**Figure 1A**). Subsequently, the leaves were harvested, and two kinds of cDNA libraries were constructed from LT-stressed and CK samples. The cDNA libraries were sequenced using Illumina technology with 10× depth. After trimming the low-quality reads, averages of 45,971,689 and 46,358,438 clean reads were obtained from the CK and LT samples, respectively. The Q30 for all sequenced libraries was greater than 95%, and the GC content of each treatment was ≥47%. Averages of about 39,943,728 (86.84%) and 40,987,248 (88.46%) clean reads were successfully mapped to the reference genome from the CK and LT samples, respectively (**Table 1**). A total of 7,9061 unigenes were procured.

**Table 1:** Summary statistics of RNA-seq generated and processed data.

Samples	Replication	Total reads	Mapped reads	Unique mapped reads	Multiple mapped reads	Pair end mapped reads	Single mapped reads	Mapped reads on positive (+) strands	Mapped reads on negative (-) strands
**Control**	Replication 1	48117818	42755427	40484781	2270646	40680430	2074997	20957374	21074499
	Replication 2	44924694	39383275	37433088	1950187	37046052	2337223	19368339	19441183
	Replication 3	44872556	37692481	34724129	2968352	35737272	1955209	18082341	18457390
	**Average of three replications**	**45971689**	**39943728**	**37547333**	**2396395**	**37821251**	**2122476**	**19469351**	**19657691**
**Low temperature stress**	Replication 1	40595778	36073778	34278941	1794837	34263206	1810572	17703661	17791797
	Replication 2	53939434	47416698	45002296	2414402	44647190	2769508	23266287	23358934
	Replication 3	44540102	39471267	37328725	2142542	37553730	1917537	19303117	19424016
	**Average of three replications**	**46358438**	**40987248**	**38869987**	**2117260**	**38821375**	**2165872**	**20091022**	**20191582**

### Annotation of the *B. napus* transcriptome

**Figure 2:**
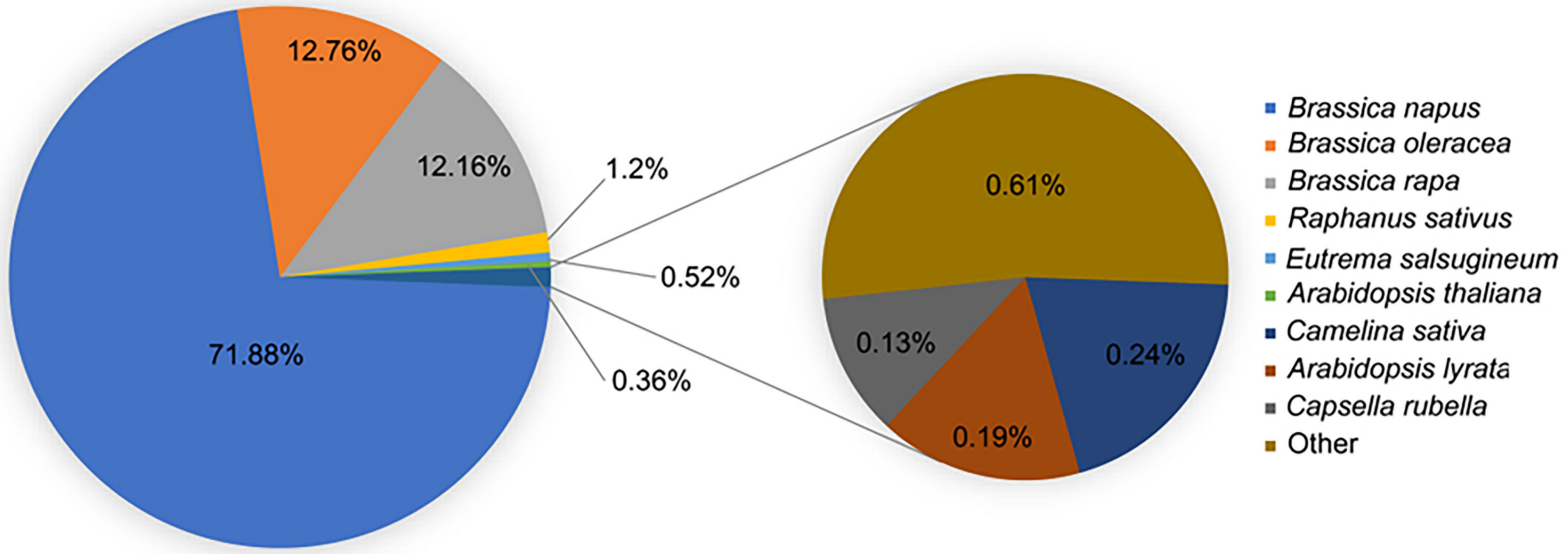
Top 10 species distribution of *B. napus* unigenes.

*B. napu* unigenes were annotated using the BLAST function against the NR and TAIR freely available databases. *B. napus* best matched with *B. napus* in the NR database compared with *B. oleracea*, *B. rapa*, *Raphanus sativus*, and other related species, which demonstrated that it was immensely homologous with *B. napus* (**Figure 2**). GO analysis was performed to categorize the function of each unigene. In total, 79,061 unigenes were successfully annotated to 55 GO terms. These terms were categorized into 55 GO terms, including 20 groups in biological processes, 14 in molecular functions, and 12 in cellular components. The biological process terms were enriched in cellular processes, metabolic processes, single organism process, responses to stimulus, and biological regulation. The enriched terms in molecular functions were binding, catalytic activity, nucleic acid binding transcription factor activity, transporter activity, and structural molecule activity. Among the cellular component terms, the five most significant classifications were cell, cell part, organelle, membrane, and organelle part (**Supplementary Figure S1**; **Supplementary Table S2**). In addition to GO analysis, the KEGG pathway analysis revealed several essential pathways involved in LT stress response. In total, 18,993 unigenes were successfully assigned to 119 pathways using KOBAS software. The major KEGG pathways were enriched in ribosome biogenesis, plant hormone signal transduction, carbon metabolism, biosynthesis of amino acids, starch and sucrose metabolism, protein processing in the endoplasmic reticulum, spliceosome, and endocytosis (**Supplementary Table S3**).

### Identification and analysis of DEGs

In order to evaluate the response to LT stress in C18, differential expression analysis was performed between samples before cytokinin (CK) and after LT treatment (LT stress). In the comparison between two groups, a total of 3,703 were identified as significant DEGs in the threshold of log_2_(fold change) ≥1 or ≤-1 (*P*-value ≤ 0.05) (**Supplementary Table S4**). To characterize the biological functions of the DEGs, we performed GO biological process (GO-BP) enrichment analysis on all upregulated and downregulated DEGs (**Supplementary Table S5**). The results showed that upregulated DEGs were mostly involved in water deprivation response, pyrimidine ribonucleotide biosynthetic process, hyperosmotic salinity response, cold response, RNA methylation, heat acclimation, chitin response, defense response, abscisic acid response, protein import into the nucleus, and response to jasmonic acid. While the downregulated DEGs mainly participated in chitin response, glucosinolate biosynthetic process, carboxylic acid catabolic process, light stimulus, and respiratory burst involved in defense response were the most enriched terms. As shown above, diverse changes and adaptations in biological reactions may occur in *B. napus* in response to LT stress.

**Figure 3:**
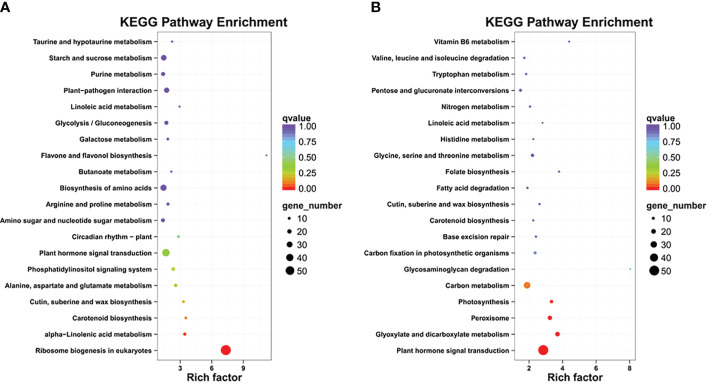
KEGG pathway enrichment: **(A)** up regulated DEGs, **(B)** down regulated DEGs.

To further categorize the important LT-stress-related pathways, the KEGG pathway enrichment analysis was performed for upregulated and downregulated DEGs (**Supplementary Table S6**). The major KEGG pathways for upregulated DEGs were ribosome biogenesis and alpha-linolenic acid metabolism, while downregulated DEGs were enriched in plant hormone signal transduction, glyoxylate and dicarboxylate metabolism, peroxisome, photosynthesis, and carbon metabolism, which suggested that LT stress has harmful effects on various biological processes, and *B. napus* simultaneously initiates its defense though complex biosynthesis and metabolic pathways (**Figures 3A, B**).

### Transcription factors’ response to LT stress

**Figure 4:**
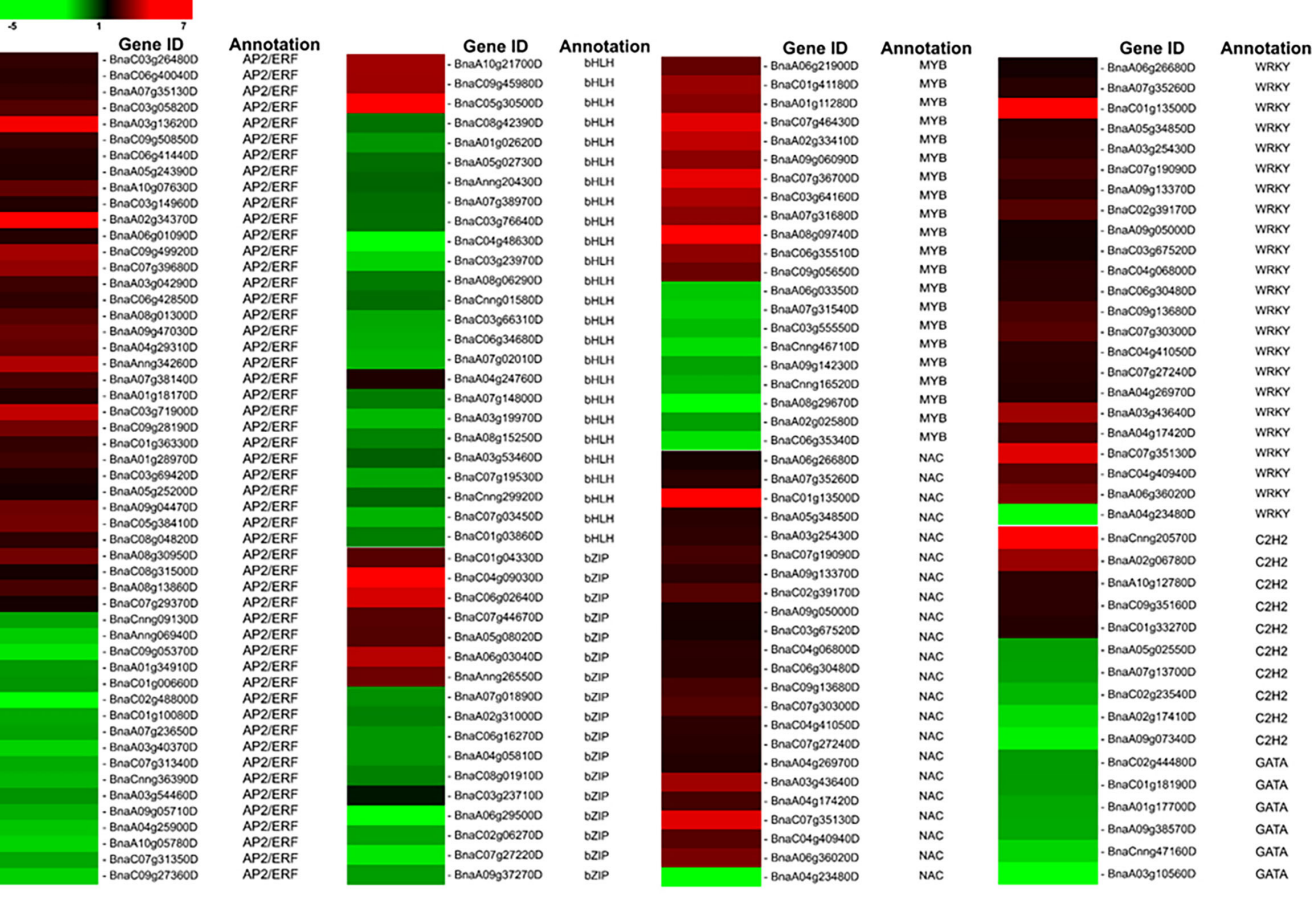
LT responsive Transcription Factors.

Transcription factors (TFs) recognize and bind to cis DNA elements—small regulatory sequences typically composed of non-coding DNA sequences (Wittkopp and Kalay, 2012)—in the promoter region to regulate the expression of downstream genes that have crucial functions in various plant stress responses. In the current study, 176 DEGs belonging to eight TF families that respond to biotic or abiotic stresses were determined (**Figure 4**). Specifically, 52 AP2/ERF members were identified, including 35 upregulated genes (*BnaC03g26480D*, *BnaC06g40040D*, *BnaA07g35130D*, *BnaA03g13620D*, *etc*.) and 17 downregulated genes (*BnaA01g34910D*, *BnaA07g23650D*, *BnaA10g05780D*, *etc*.). In our data, the basic helix–loop–helix (bHLH) family was the second largest that consist of 25 members, including three upregulated genes (*BnaA10g21700D*, *BnaC09g45980D*, and *BnaC05g30500D*) and 22 downregulated genes. We detected 21 MYB family members, including 12 upregulated and nine downregulated genes that respond to LT stress in *B. napus*. In the current study, it is also evident that WRKY TFs play significant regulatory roles in *B. napus* under LT stress (**Figure 4**). We found that 23 WRKY TFs respond to LT stress; among them, 22 members have induced expression, and only one member, *BnaA04g23480D*, was repressed under LT stress. Similarly, a total of 22 NAC TFs were found in this study, including 19 upregulated members and two downregulated members (*BnaCnng30500D* and *BnaA04g24740D*). In addition, among all identified basic leucine zipper transcription factor (bZIP TF) family members, seven were upregulated, while 11 were downregulated. Thus, it is suggested that the strong activation or repression of transcription factors by LT stress may promote the defense response in *B. napus*.

### Analysis of DEGs related to potential pathways

**Figure 5:**
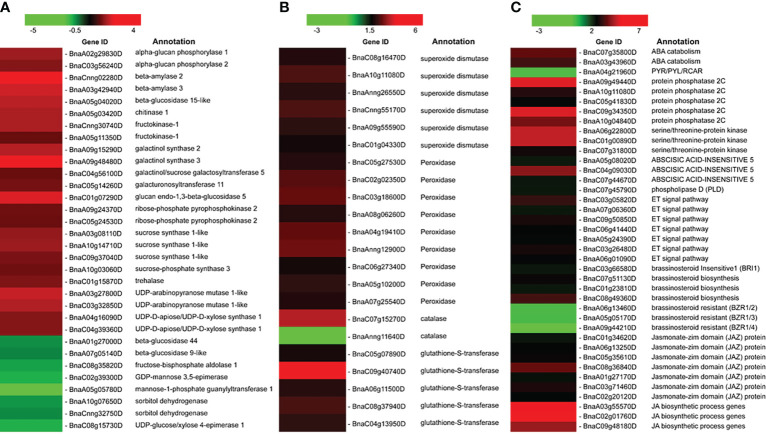
Heatmap of DEGs in RNA-seq data related to: **(A)** Sugar, **(B)** Antioxidant, **(C)** Phytohormone.

When plants were exposed to cold stress, dynamic changes in starch content occurred. Many genes involving starch and sucrose metabolism have been previously proven to respond to LT stress, such as ADP-glucose pyrophosphorylase, β-amylase, and sucrose synthases, and were detected (Sicher, 2011; Yu et al., 2022a). As summarized in the heat map (**Figure 5**), different combinations of starch-degrading enzymes operate under LT stresses, and most of them were upregulated—for instance, we found the activation of three genes encoding sucrose synthase 1-like (*BnaA03g08110D*, *BnaC09g37040D*, and *BnaA10g14710D*) that promotes the catabolization of sucrose to uridine di-phosphoglucose and fructose. Various genes involved in galactose metabolism were also upregulated, such as *BnaA09g15290D*, *BnaA09g48480D*, and *BnaC04g56100D*, which encode galactinol synthase 2, galactinol synthase 3, and galactinol/sucrose galactosyltransferase 5. These results are consistent with previous reports (Ciereszko et al., 2001; Baud et al., 2004). ROS such as hydrogen peroxide (H_2_O_2_), superoxide (O^2-^), singlet oxygen (^1^O_2_), and hydroxyl radical (OH^-^) are quickly produced from multiple organelles under LT stress (Qi et al., 2018), known as toxic agents, and also perceived as the second messengers by plant cells and trigger rapid responses. Here almost all DEGs belonging to the ROS-scavenging enzyme system were upregulated, including six SOD members, one CAT member, nine POD members, and five glutathione S-transferase (GST) members (**Figure 5**), except only one CAT gene (*BnaAnng11640D*) that was downregulated, suggesting that these enzymes were actively expressed to form a complex antioxidant defense under short-time LT stress. Phytohormones include auxins, ABA, gibberellins (GA), CK, ethylene (ET), salicylic acid, jasmonates (JA), brassinosteroids (BR), and strigolactones, and they play critical roles in helping the plants to adapt to temperature stresses (Verma et al., 2016). As shown in **Figure 5**, 14 DEGs involved in the ABA signal pathway have an induced expression in response to LT stress, followed by one gene that showed a repressed expression. In addition, seven ET signal pathway-related and 10 JA signal pathway-related DEGs were upregulated. Three DEGs belonging to the BR signal pathway were significantly downregulated; however, three BR biosynthesis pathway genes were upregulated. Therefore, the abovementioned results indicated that the ABA, ET, BR, and JA signaling pathways were activated in the LT stress response of *B. napus*.

### Interaction network between *B. napus* LT-stress-related genes

**Figure 6:**
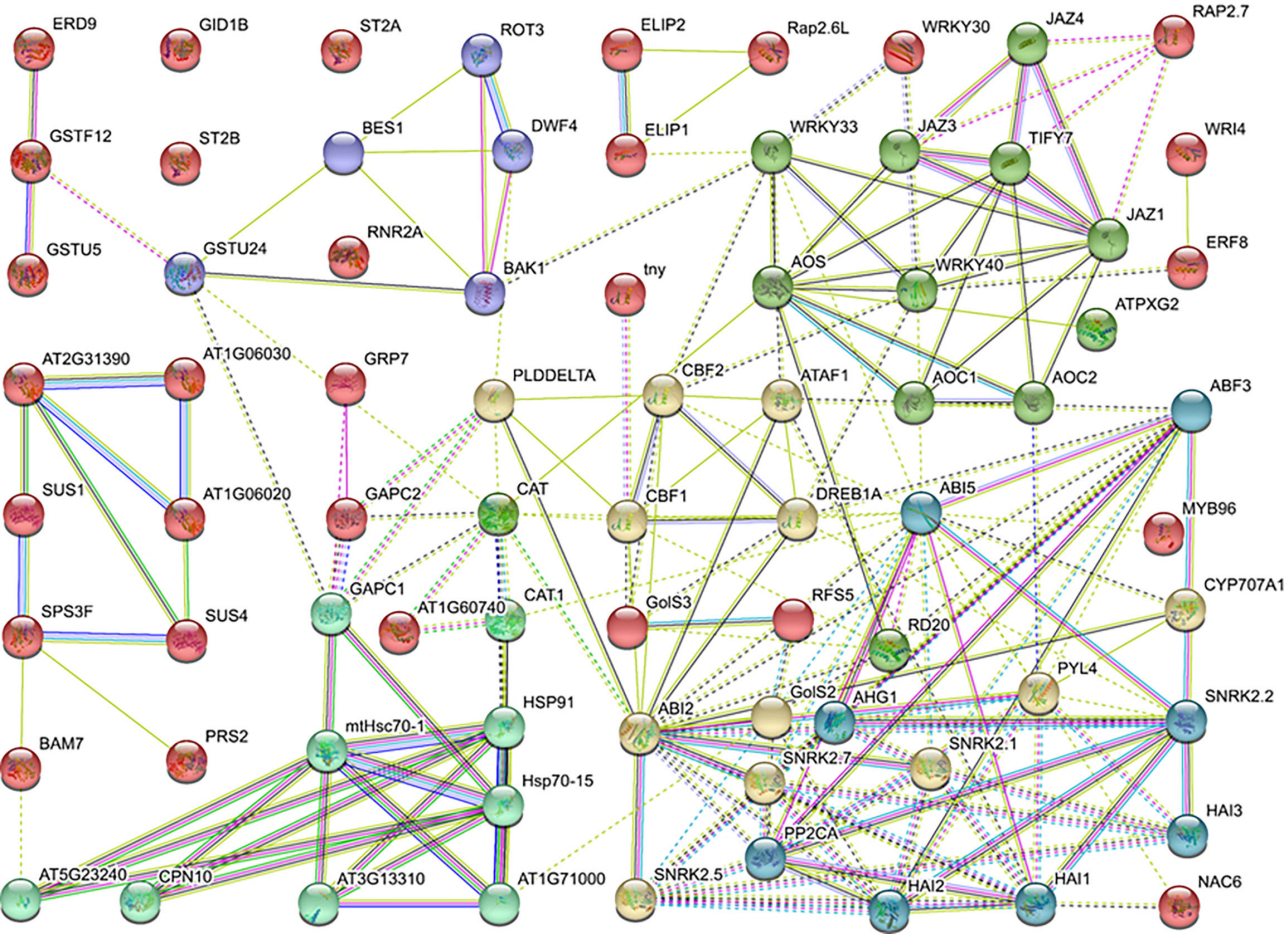
Protein-protein interaction network.

To elaborate the genes’ role in regulating LT response at the protein level, the protein–protein interactions network was constructed using DEGs enriched in GO or KEGG terms, including sugar metabolism, hormone, antioxidant activity, transcription factors, and photosynthesis. The homologs of potential DEGs were referred to the *Arabidopsis* database and subjected to STRING database analysis. As the results demonstrated, the homologs of DEGs were divided into six expression patterns, and the interaction lines exhibited different types of association among genes. Specifically, PYR/PYL/RCAR-PP2C-SnRK2-ABF, known as the ABA-dependent pathway, was detected. Meanwhile, nearly half of the genes were associated with each other and regulated the CBFs and corresponding stress-related genes. Besides the abovementioned data, JAZ/TIFYs pathway genes were detected, which indicates that the jasmonic acid signal pathway may participate in the quick cold response of *B. napus* (**Figure 6**).

### Validation of transcriptome data by qRT-PCR analysis

**Figure 7:**
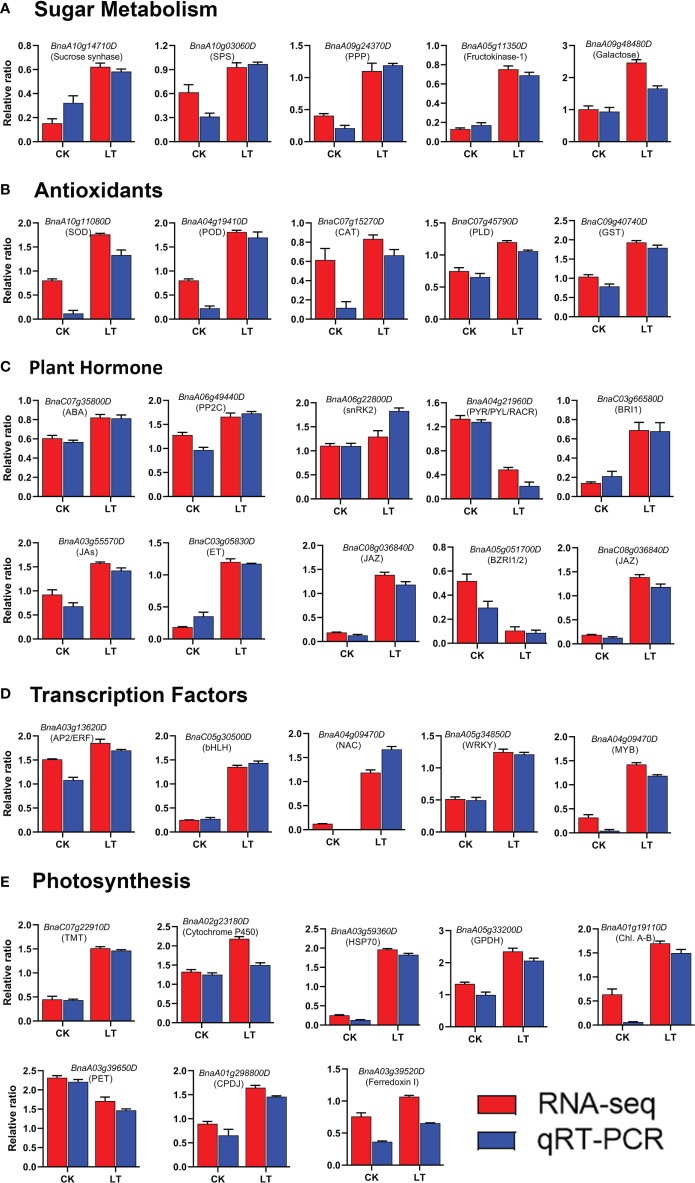
qRT-PCR validation of RNA-seq data: **(A)** Sugar metabolism, **(B)** Antioxidants, **(C)** Plant Hormone, **(D)** Transcription factors, **(E)** Photosynthesis.

To validate the transcriptome data, 32 candidate genes were randomly selected and subjected to qRT-PCR analysis. These target genes belong to various functional categories of the KEGG pathways, including photosynthesis, sugar metabolism, plant hormone signal transduction, anti-oxidant defense systems, and transcription factors. Finally, we compared the RNA-seq expression level with the qRT-PCR results. The qRT-PCR results were consistent with the RNA-seq, confirming the reliability of the transcriptome results (**Figure 7**).

## Discussion

To dissect the LT response mechanisms in *B. napus*, various LT-related candidate DEGs were explored from transcriptomic data, and their mRNA abundance was investigated in LT stress response processes. In this study, our primary focus was on those DEGs involved in sugar metabolism (starch, sucrose, and carbohydrate), photosynthesis, plant antioxidant defense system, plant hormone signaling networks, and TFs. The LT stress had adverse effects on photosynthesis. Under LT stress, the cellular respiration process of mitochondria and the photosynthesis process in chloroplast generate oxidative stress and lead to ROS stockpiling (Oelze et al., 2008; Janmohammadi et al., 2015). Oxidative stress due to higher levels of ROS accumulation causes damage to DNA, lipids, and proteins (Schieber and Chandel, 2014). In *B. napus*, photosynthesis-related genes were mostly downregulated and took part in the light reaction and the Calvin cycle. Decreased photosynthesis activity is directly linked with reduced plant productivity under LT stress conditions. In addition to downregulated genes, few genes were induced and might have played a significant role in protecting the photosynthesis system under LT stress in *B. napus*. Genes involved in phytohormone signaling networks, sugar metabolism, and antioxidants had variable expression levels, which showed the complexity of the molecular regulation network in response to LT stress (Bari and Jones, 2009; Li et al., 2016a; Ding et al., 2019).

### Sugar metabolism, source of energy, and keeping osmotic homeostasis

Previous studies demonstrated that soluble sugars accumulate in response to stress; however, they may play multiple roles under stress. Sugar molecules act as biochemical components for cold acclimation and protect plant cells from damage (Gilmour et al., 2000). Sugars also function as stress signal molecules and trigger a series of signal transduction and defense reactions (Eveland and Jackson, 2012). Sucrose synthase and sucrose phosphate synthase are essential enzymes responsible for metabolic energy in the sugar metabolism pathway (Fugate et al., 2019; Stein and Granot, 2019). During LT stress, Pi accumulation decreased in the cytoplasm (Strand et al., 1999), and altered Pi level signals led to the activation of enzymes in the sucrose synthesis pathway (Hurry et al., 2000). Thus, we have focused on the starch and sucrose metabolism pathway in *B. napus* and several genes related to sugar metabolism surveyed under LT stress conditions (**Figure 5**) and verified their expression pattern through qRT-PCR (**Figure 7**). Different kinds of sugars accumulated in plants, such as sucrose, glucose, raffinose, and fructose, which are involved in LT tolerance (Gu et al., 2018). Sucrose synthase involved in sucrose catabolism and sucrose phosphate synthase involved in biosynthesis are important enzymes primarily responsible for metabolic energy in the sugar metabolism pathway. The sucrose catabolization process acts as a source of ATP or cell wall biosynthesis (Fugate et al., 2019). Therefore, sucrose synthase enzymes are mainly localized in cell walls, mitochondria, and vacuoles (Stein and Granot, 2019). Similarly, upregulated sucrose synthase genes were reported under LT stress in *Arabidopsis* (Ciereszko et al., 2001; Baud et al., 2004). Furthermore, one sucrose phosphate synthase gene (*BnaA10g03060D*) was upregulated, which describes the possible role of sucrose biosynthesis under LT stress in *B. napus* (**Figure 5A**). Similarly, our biochemical analysis revealed the higher accumulation of soluble sugar and proline under LT stress (**Figures 1F, G**). A previous study has reported that up to 50% of the activity of sucrose phosphate synthase gene was induced in wheatgrass under LT stress (Jaikumar et al., 2016). In rice, the interaction of calcium-dependent protein kinase OsCPK17 with sucrose phosphate synthase and plasma membrane intrinsic protein is compulsory for LT stress response (Almadanim et al., 2017). Fructokinase plays an essential role in sugar metabolism, signaling, and growth and also has osmosis-protective effects in LT stress (Li et al., 2017b). A high expression of fructokinase genes has been proven to enhance LT tolerance in *Arabidopsis* (Su et al., 2018). In this study, two genes encoding fructokinase-1 (*BnaCnng30740D* and *BnaA05g11350D*) were upregulated in *B. napus* under LT stress (**Figure 5A**). Activation of these fructokinase genes suggested a critical role in LT stress tolerance in *B. napus*.

The overexpression of galactinol synthase (GolS) genes enhanced LT stress tolerance in *Ammopiptanthus nanus* (Liu et al., 2016). GolS catalyzes the raffinose family oligosaccharide biosynthetic pathway (Salvi et al., 2016). In the current study, various genes involved in galactose metabolism were also upregulated (**Figure 5A**). Similarly, pentose phosphate pathway genes were induced, such as *BnaA09g24370D* and *BnaC05g24530D* which encode ribose-phosphate pyrophosphokinase 2, which was chloroplastic under LT stress (**Figure 5A**). It has been reported that pentose phosphate pathway genes are hub genes involved in rapid rapeseed seed germination under cold stress (Luo et al., 2019). We also observed multiple up- and downregulated genes, such as beta-D-xylosidase 4, involved in amino sugar and nucleotide sugar metabolism. Hence, when exposed to LT stress, numerous genes involved in sucrose–starch metabolism were induced or repressed, which may lead to the accumulation of osmoprotectants such as sucrose, glucose, and fructose, subsequently maintaining osmotic protection or providing necessary energy under LT stress conditions.

### Antioxidant defense system: A cellular cushion against LT stress

A higher accumulation of ROS after LT stress always causes oxidative damage to cellular organs, such as nucleic acid, lipids, and proteins (Suzuki and Mittler, 2006; Schieber and Chandel, 2014). Accordingly, we observed higher H_2_O_2_ levels in LT-stressed *B. napus* seedlings (**Figure 1D**). In addition to oxidative damage, ROS-mediated activated enzymatic and non-enzymatic ROS scavenging systems detoxify and relieve the cellular oxidative stress level (Miller et al., 2010; Sies et al., 2017). In *B. napus* during LT stress, the ROS-scavenging enzymatic antioxidant system consists of POD, SOD, CAT, APX, GST, and glutathione peroxidase (GPX) (Yan et al., 2019). The non-enzymatic antioxidant systems included the reduced form of glutathione system, vitamin E\vitamin C system, and secondary metabolites or antioxidants, such as carotenoids, steroids, flavonoids, and anthocyanins. In *B. napus*, ROS homeostasis—by utilizing these antioxidant systems—protects cellular oxidative stress injury. SOD is known as the first line of defense for ROS-scavenging systems. SOD converts damaging free radicals to less harmful products (H_2_O_2_ and O_2_) in cells through the dismutation process (Zhang et al., 2019b). The induced expression of SOD genes upon cold exposure was reported in *Medicago truncatula* (Song et al., 2018) and *Setaria italica* (Wang et al., 2018b). In plants, SODs are mainly divided into three classes based on metal co-factors: copper- and zinc-containing superoxide dismutase (Cu/Zn-SODs), iron superoxide dismutase (Fe-SOD), and manganese superoxide dismutase (Mn-SOD). Different classes of SODs are distributed at different positions or in organs in the cell. Cu/Zn-SODs are generally considered eukaryotic enzymes mainly distributed in the cytosol and chloroplast (Kroll et al., 1995; Wang et al., 2018a). Our findings indicate that various SOD genes (*BnaC08g16470D*, *BnaA10g11080D*, *BnaAnng26550D*, *BnaCnng55170D*, *BnaA09g555*90D, and *BnaC01g04330D*) were induced under LT stress conditions, indicating that it may have LT response regulation in *B. napus* (**Figure 5B**). Similarly, we observed an induced SOD activity in LT-stressed seedlings through biochemical analysis, which indicates that SOD activity is genetically controlled (**Figure 1H**). POD and CAT catalyzed H_2_O_2_ into simple H_2_O in cells. Ascorbate peroxidase enzymes detoxify H_2_O_2_ in plant cells. APX is involved in the ascorbate–glutathione cycle to catalyze the H_2_O_2_ into H_2_O using ascorbate as a specific electron donor. APX enzymes are mainly located in subcellular compartments, such as chloroplasts, cytosol, mitochondria, and peroxisome (Caverzan et al., 2012). In our results, the higher activation of APX enzyme activity depicted a protective role from the accumulated H_2_O_2_ and MDA by converting them into less harmful components or exclusion from the cellular environment (**Figures 1D, E**). In this study, POD genes (*BnaC05g27530D*, *BnaC02g02350D*, *BnaC03g18600D*, *BnaA08g06260D*, *BnaA04g19410D*, *BnaAnng12900D*, *BnaC06g27340D*, *BnaA05g10200D*, and *BnaA07g25540D*) were significantly upregulated upon LT stress exposure in *B. napus*. Accordingly, our biochemical analysis revealed the induction of POD activity in LT-stressed *B. napus* seedlings, which indicates a close relationship between the expression of these POD genes and POD enzyme activity (**Figures 1**, **5B**). One of the CAT genes’ expressions (*BnaC07g15270D*) was upregulated, while another (*BnaAnng11640D*) was repressed. POD and CAT genes have variable responses to the LT stress, which implicated that these genes may play divergent roles during LT stress response in *B. napus* (**Figure 5B**). We found that CAT enzyme activity was suppressed in LT stress seedlings (**Figure 1K**). These results also indicate that rapeseed A genome genes might play a critical role in the CAT enzyme activity compared with the B genome. Similarly, GST and GPX respond to environmental stress and act as scavengers of ROS and oxidizing radicals (Milla et al., 2003; Xu et al., 2015). In this study, the expression of GST genes (*BnaC05g07890D*, *BnaC09g40740D*, *BnaA06g11500D*, *BnaC08g37940D*, and *BnaC04g13950D*) was significantly enhanced, depicting their role in LT stress (**Figure 5B**). In one sentence, these findings elaborated various ROS signaling pathways and provided insight into antioxidant genes, which formed a complex antioxidant defense system under LT stress conditions in *B. napus*.

### Plant hormone signal network to encounter LT stress

**Figure 8:**
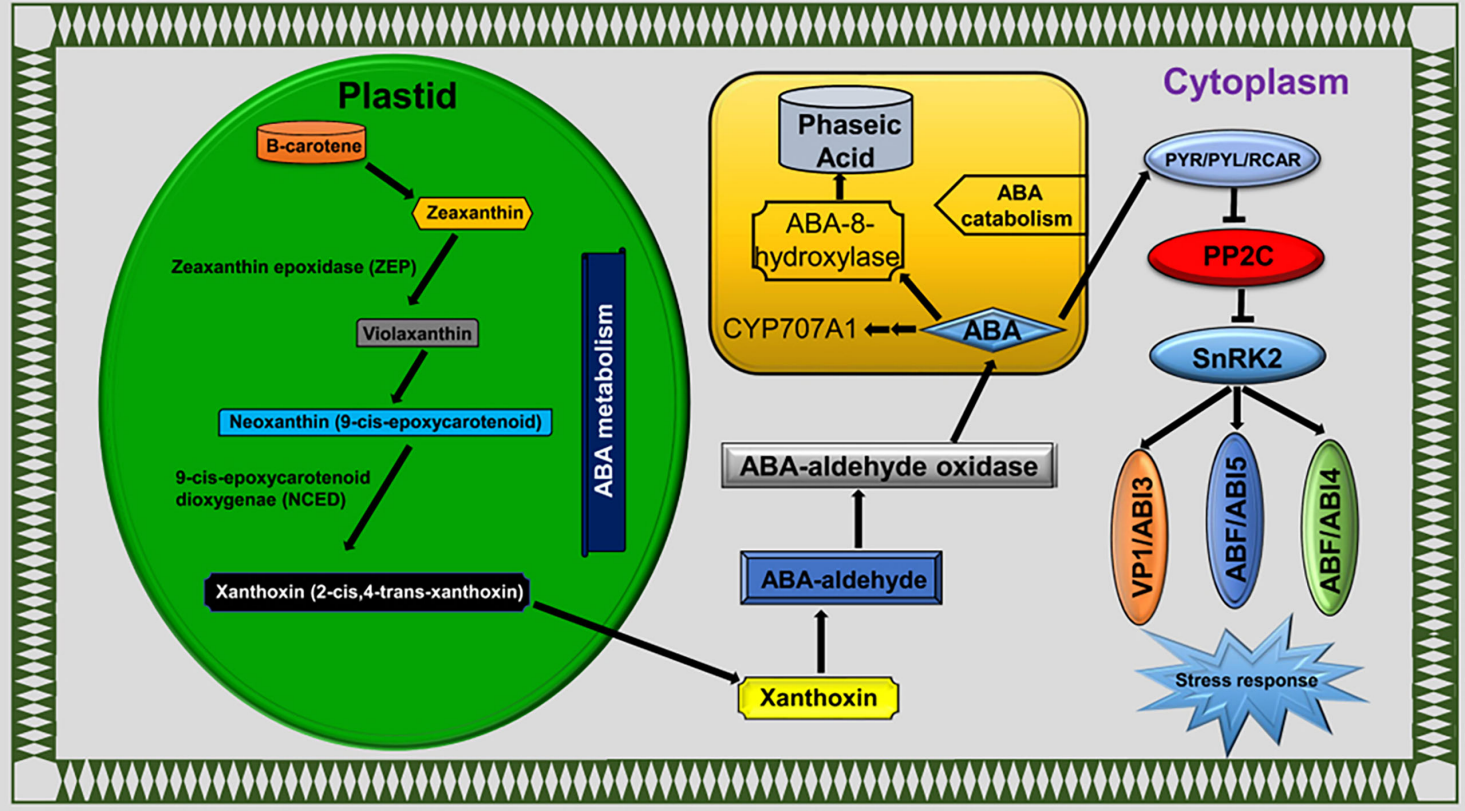
ABA pathway.

Plant hormones were extensively studied under various environmental cues, including LT stress conditions in plants. Phytohormones have key functions in stress responses, such as to initiate a series of signal events and activate the expression of stress-responsive genes (Ku et al., 2018). Calcium sensors in cellular membranes respond to external stimuli to activate downstream signaling events and are influenced by ABA (Edel and Kudla, 2016). The plant responds to LT stress *via* ABA-dependent and ABA-independent pathways (Gusta et al., 2005; Ishitani et al., 1997). ABA accumulation mitigates the LT stress effects in rice (Zhao et al., 2015) and bermudagrass (Huang et al., 2017). Interestingly, exogenous ABA treatment reduced the H_2_O_2,_ electrolyte leakage and MDA contents in bermudagrass compared with non-ABA-treated plants under LT stress conditions (Huang et al., 2017). In this study, we detected the ABA biosynthesis, catabolism, and signaling pathway genes as DEGs (**Figure 8**). Our findings indicated that genes involved in ABA catabolism, such as CYP707A1/2 (*BnaC07g35800D* and *BnaA03g43960D*), were activated in response to LT stress in *B. napus* (**Figure 5C**). CRY707A encodes cytochrome P450 monooxygenase that catalyzes the ABA into 8′-hydroxy ABA (Seiler et al., 2011). During the biosynthesis process, ABA repressed the expression of CYP707A genes but increased the expression of NCED. Variation in CYP707A expression level changed the cellular ABA levels and accordingly repressed or induced the NCED expression level. ABA catabolism and biosynthesis work antagonistically, which shows a strong feedback and feedforward loop mechanism to limit or enhance the ABA contents in cells. In addition to ABA accumulation, CYP707A works as a hub to coordinate with auxin, GA, and ABA signals (Liao et al., 2018). The variable expression level of CYP707A genes suggested that ABA catabolism and biosynthesis work side by side in response to LT stress in *B. napus* (**Figure 4**). The ABA signaling pathway is a double-negative regulatory system consisting of proteins SNF1-related protein kinase 2 (SnRK2), type 2C protein phosphatase (PP2C), and ABA receptors pyrabactin resistance 1 (PYR1)/PYR1‐like (PYLs)/regulatory components of ABA receptors (RCAR) family (Hauser et al., 2011). In the absence of ABA, PP2C-mediated dephosphorylation repressed SnRK2 kinases to block signal transduction. In response to external stimuli, ABA-receptor PYR/PYL/RCAR protein inactivates PP2C, resulting in SnRK2 activation. Activated SnRK2 causes the upregulation of bZIP group TFs to increase the mRNA level of downstream ABA-responsive genes (Sirichandra et al., 2009; Li et al., 2011) (**Figure 8**). ABA-insensitive 5 (ABI5) is a bZIP TF that regulates ABA-dependent seed germination, growth, and development under environmental constraints. ABI regulates the expression of downstream ABA pathway genes that consist of the ABSCISIC ACID RESPONSE ELEMENT motif in the promoter region (Skubacz et al., 2016). The *Arabidopsis* mutant for *ABI5* genes exhibited a reduced ABA level and seed dormancy, while complementation of *ABI5* rescues the expression of ABA-responsive genes and dormancy. Similarly, the exogenous application of ABA reduces the cold sensitivity of the *ABI* mutant of *Arabidopsis* (Wu et al., 2015). Phospholipase D (PLD) involved in ABA signaling is a key regular of plant growth, development, and abiotic stress in *B. napus* (Lu et al., 2019). In this study, PYR/PYL/RCAR (*BnaA04g21960D*) was significantly downregulated. Meanwhile, PP2C (*BnaA09g49440D*, *BnaA10g04840D*, *BnaA10g11080D*, *BnaC05g41830D*, and *BnaC09g34350D*), SnRK2 (*BnaA06g22800D*, *BnaC01g00890D*, and *BnaC07g31800D*), ABI5 (*BnaA05g08020D*, *BnaC04g09030D*, and *BnaC07g44670D*), and PLD gene (BnaC07g45790D) were found to be upregulated under LT stress (**Figure 5C**). These findings suggested that the ABA signaling pathway actively responds to LT stress in *B. napus*.

The phytohormone ethylene (ET), C_2_H_4_, is an important regulator of the cold stress response. It is debated whether the effect of ET response to LT stress is negative or positive in plants (Hu et al., 2016). ET negatively affects freezing tolerance as the repression of ET biosynthesis enhances freezing tolerance in *Medicago truncatul*a seedlings (Zhao et al., 2014). On the other side, ET accumulation positively regulates cold tolerance in grapevine (Sun et al., 2016). These results indicate that ET accumulation imparts LT stress tolerance in species-specific manners. The mechanism of the ethylene signaling pathway from ethylene recognition at the cellular membrane to transcriptional regulation in the nucleus and the post-transcriptional level was well reported (Yang et al., 2015; Ku et al., 2018). CTR1 modulates the ethylene signaling pathway through a direct interaction with ET receptors. Active CTR1 can repress EIN2, but EIN2 repression is rescued in the presence of ET. The EIN2 translocates from the cell membrane and activates EIN3/EIL1 in the nucleus. EIN3 and EIL1 are key TFs that modulate ET-responsive gene expression (Ju et al., 2012). The EIN3 targets the ETHYLENE RESPONSE FACTOR1 in *Arabidopsis*. The ubiquitin/proteasomal degradation system regulated the EIN3 and EIL1 levels in *Arabidopsis*. EIN2 is compulsory for mediating ethylene-induced EIN3/EIL1 expression and EIN3-binding F-box protein (EBF1/2) degradation (An et al., 2010). We observed several ET signal pathway-related genes—*BnaC03g05820D*, *BnaA07g06360D*, *BnaC09g50850D*, *BnaC06g41440D*, *BnaA05g24390D*, *BnaC03g26480D*, and *BnaA06g01090D*—which were differentially expressed and may participate in *B. napus* LT stress response (**Figure 5C**).

Plant steroids such as brassinosteroids (BR) bind to protein receptors in membranes, regulate membrane structure and growth, and enhance abiotic stress tolerance (Filek et al., 2017). Brassinosteroids enhanced cold stress tolerance in *Medicago truncatula* (Arfan et al., 2019). In this study, genes related to BR signal pathways were deduced, such as brassinosteroid insensitive 1 (BRI1) precursor (*BnaC03g66580D*), brassinosteroid biosynthesis (*BnaC07g51130D*, *BnaC01g23810D*, and *BnaC08g49360D*), and brassinosteroid resistant (BZR1/2) (*BnaA06g13460D*, BnaA05g05170D, and *BnaA09g44210D*), which were differentially expressed in LT-stressed *B. napus* (**Figure 5C**). BRI1 is a transmembrane receptor kinase. BRI1 is required for BR perception at the cell membrane surface and signal transduction *via* phosphorylation of brassinosteroid signaling kinase. BIN2 phosphorylates BZR1 and BZR2 to downregulate the BR-responsive genes (Zhang et al., 2014). In *Arabidopsis*, overexpression of BZR1 and knockdown of *BIN2* genes enhanced the freezing tolerance *via* CBF-dependent and CBF-independent pathways (Li et al., 2017a).

Similarly, the phytohormone JA signaling pathway played a key role in signaling and activating the downstream CBF/DREB1 pathway during cold stress in *Arabidopsis* (Zheng et al., 2018). Exogenous JA effectively increased the freezing tolerance of *Artemisia annua* (Liu et al., 2017b). Various genes encoding the Jasmonate-zim domain (JAZ) protein (*BnaC01g34620D*, *BnaA06g13250D*, *BnaC05g35610D*, *BnaC08g36840D*, *BnaA01g27170D*, *BnaC03g71460D*, and *BnaC02g20120D*) were induced under LT stress in this study (**Figure 5C**). The JAZ proteins bind to the promoter’s region of TFs, such as MYC, to repress jasmonate signaling (**Figure 6**) (Katsir et al., 2008). CORONATINE INSENSITIVE 1 (COI1) works as a JA receptor and is an important part of the SCF–CoI1 ubiquitin E3 ligase complex. COI1 used the ubiquitin/proteasome pathway to degrade the JAZ protein. JA biosynthetic process genes (*BnaA03g55570D*, *BnaC02g01760D*, and *BnaC09g48180D*) are activated under LT stress (**Figure 5C**). These results implied that jasmonate signal pathways might function in *B. napus* LT response processes. In a nutshell, the LT stress response of *B. napus* variety C18 was a complicated network involving the ABA, JA, BR, and ET signal pathways.

### Transcription factor: efficient regulators of genomes in response to LT stress

Different TF families played an important role in enhancing LT stress tolerance, such as APETALA2/ethylene response factor (AP2/ERF), bZIP, WRKY, and MYB in plants. The AP2/ERF TF family gene members are important regulators in stress response (Mizoi et al., 2012). In a previous study, 132 AP2/ERF TFs were found to differentially express and respond to LT stress in rapeseed (Du et al., 2016). The bHLH TF family is the second largest protein family in plants that respond to multiple stresses including drought, salt, and especially cold stress (Sun et al., 2018; Li et al., 2020). MYB TFs exhibited diverse roles in growth, development, anthocyanin accumulation, hormone responses, and cold stress tolerance (An et al., 2020). WRKY TFs played an active role in plant defense against biotic and abiotic challenges. The regulation of WRKY TF enhanced cold tolerance in grapevine, and the heterologous expression of WRKY enhanced *Arabidopsis*’ cold tolerance (Sun et al., 2019). A significant induction of WRKY TFs demonstrates the prominent roles of this family in *B. napus* LT stress response (**Figure 4**). Similarly, NAC TFs engaged in multiple biological processes, including signal transduction, development, and biotic and abiotic stress responses. The plant cold stress response of the NAC TF family was evident in *Prunus mume* (Zhuo et al., 2018), pepper (Hou et al., 2020), and rice (Pradhan et al., 2019). In previous studies, the role of bZIP TFs in cold stress tolerance was described, such as in *B. oleracea* (Hwang et al., 2016), *Magnolia wufengensis* (Deng et al., 2019), and rice (Pradhan et al., 2019). In addition, other TFs such as C_2_H_2_, G2-like, GATA, HSF, Trihelix, and ZF-HD, either up- or downregulated, were found in response to LT stress in *B. napus*. The relative expression of LT-stress-responsive TFs was verified by qRT-PCR (**Figure 7**). The differential expression of numerous TFs suggested that relatively complex networks activated in response to cold stress in *B. napus* are regulated at the transcriptional level to fine-tune various biological pathways to enhance LT stress tolerance.

### Role of photosynthesis system in LT stress response

The chloroplast, a photosynthesis organ, is acutely sensitive to LT stress. Thus, LT stress halts the photosynthesis process in plants (Ma et al., 2018). Photosystem II (PSII) is a crucial component of a photosynthetic machinery that is actively inactivated under stress, and the accumulation of ROS halts the repairing mechanism of PSII in plants (Nishiyama and Murata, 2014). LT stress reduces the photosynthetic capacity by affecting the electron transfer in PSII and the efficiency of CO_2_ fixation to photosynthates (Paredes and Quiles, 2015). The repair mechanism of PSII involves the replacement of damaged D1 protein by newly synthesized D1 protein at the expense of ATP in the degradation and synthesis of D1 (Murata and Nishiyama, 2018). In this study, primarily genes related to the photosynthesis pathway were repressed, which indicated that LT stress halts the photosynthesis system in *B. napus* (**Table 2**). Accordingly, we also found that the maximum quantum efficiency of PSII Fv/Fm was reduced in LT-stressed seedlings, indicating a direct relationship with photosynthesis pathway genes (**Figure 1C**). Most of these genes are essential components of the light reaction and the Calvin cycle process of photosynthesis. During light reaction, sunlight was utilized by chlorophyll pigments to synthesize the high-energy compounds ATP and NADPH (Armbruster et al., 2017). The Calvin cycle is a light-independent redox reaction to fix carbon dioxide into the sugar glucose molecules and gaseous detoxification during photosynthesis (Sun et al., 2015; Nowicka and Kruk, 2018). Repression of genes involved in photosynthetic components were reported in tea (Shi et al., 2019), hibiscus (Paredes and Quiles, 2015), and rice (Liu et al., 2018b) under LT stress. Nonetheless, we also observed that numerous genes in the photosynthetic apparatus were induced during LT stress in *B. napus*. Among upregulated genes, genes were related to chlorophyll A–B-binding protein (*BnaC01g22830D*, *BnaA01g19110D*, and *BnaA01g24440D*), cytochrome P450, thylakoid membrane, photosynthetic reaction center protein (*BnaC09g27520D*), and ferredoxin I (*BnaC07g49690D* and *BnaA03g39520D*), while genes related to photosynthetic electron transport (*BnaA03g39650D*) were downregulated. Besides the abovementioned details, two gene families like chloroplastic chaperone protein dnaJ (*BnaA01g29880D* and *BnaA02g15280D*) and heat shock protein 70 (HSP70) members (*BnaC03g61170D*, *BnaA03g59360D*, and *BnaA06g00870D*) were induced. Under stress, DnaJ proteins function for protein homeostasis and protein complex stabilization. Transgenic tomato plants overexpressing DnaJ proteins exhibited maximum efficiency and stability of PSII and D1 protein complexes under cold stress conditions. During cold stress, HSP70 was discovered as the partner of DnaJ proteins (Kong et al., 2014). Ferredoxin molecules are sensitive to stress. Engineering of tobacco chloroplasts by an isofunctional protein of ferredoxin (a cyanobacterial flavodoxin) enhanced the tobacco plant tolerance to multiple stresses, including chilling (Zurbriggen et al., 2008). In our study, some genes related to glyceraldehyde-3-phosphate dehydrogenase (GAPDH)—such as *BnaA0*5g33200D, *BnaC05g47450D*, and *BnaC01g40210D*—were induced (**Table 2**). GAPDH is involved in cellular metabolism and generates energy. GAPDH was induced in potato tubers under cold stress (Liu et al., 2017a). The ubiquitous enzyme adenylate kinase interacts with the chloroplast GAPDH, forming a stable complex that regulates the ATP/NADPH ratio inside the chloroplast to optimize the Calvin–Benson cycle in response to environmental stress (Zhang et al., 2018). Then, we performed qRT-PCR to verify the expression pattern of photosynthesis pathway-related DEGs, which further confirmed the reliability of the RNA-seq data (**Figure 7**). Thus, differential expression of photosynthetic system genes indicates that they may play vital roles in response to LT stress in *B. napus*. The exact mechanism of these factors remains unresolved and provides new directions for future LT stress studies.

**Table 2:** Photosynthesis pathway genes.

B. Process	Gene ID	Arabidopsis	Annotation	Regulation
**Light reaction**	BnaC01g22830D	AT4G14690.1	Chlorophyll A–B binding protein	up
	BnaCnng14300D	AT3G22840.1	Chlorophyll A–B binding protein	up
	BnaC03g43360D	ATCG00065.1	Photosystem I psaA/psaB protein	down
	BnaA02g23180D	AT5G42650.1	Cytochrome P450	up
	BnaA01g19110D	AT4G14690.1	Chlorophyll A–B binding protein	up
	BnaC04g15570D	ATCG00350.1	Photosystem I psaA/psaB protein	up
	BnaC09g30700D	AT5G54270.1	Chlorophyll A–B binding protein	down
	BnaC09g27520D	ATCG00270.1	Photosystem II protein	up
	BnaA03g36810D	AT3G22840.1	Chlorophyll A–B binding protein	up
	BnaA05g29390D	AT5G01530.1	Chlorophyll A–B binding protein	down
	BnaCnng37300D	AT3G22840.1	Chlorophyll A–B binding protein	up
	BnaC06g20460D	AT3G22840.1	Chlorophyll A–B binding protein	up
	BnaA01g24440D	AT3G22840.1	Chlorophyll A–B binding protein	up
	BnaA02g34030D	AT5G64040.2	Photosystem I reaction center subunit N	down
	BnaA08g01590D	AT1G52230.1	Photosystem I reaction center subunit VI	down
	BnaC03g69350D	AT1G52230.1	Photosystem I reaction center subunit VI	down
	BnaC03g59670D	AT1G30380.1	Photosystem I psaG/psaK	down
	BnaC09g06210D	AT5G64040.2	Photosystem I reaction center subunit N	down
	BnaC01g12690D	AT4G21280.2	Oxygen evolving enhancer protein 3	down
	BnaA01g11190D	AT4G21280.2	Oxygen evolving enhancer protein 3	down
	BnaC05g10420D	AT1G14150.1	Oxygen evolving enhancer protein 3	down
	BnaCnng05610D	AT3G01440.1	Oxygen evolving enhancer protein 3	down
	BnaA06g08980D	AT1G14150.1	Oxygen evolving enhancer protein 3	down
	BnaAnng20940D	AT5G49730.1	Photosynthetic electron transport chain	down
	BnaA07g24840D	AT1G68010.2	Photosynthetic electron transport chain	down
	BnaA07g26770D	AT1G68010.2	Photosynthetic electron transport chain	down
	BnaA03g39650D	AT5G23060.1	Photosynthetic electron transport chain	down
	BnaC06g29160D	AT1G68000.1	Photosynthetic electron transport chain	down
	BnaC09g26410D	AT5G49730.1	Photosynthetic electron transport chain	down
	BnaA01g28110D	AT3G16250.1	Photosynthetic electron transport chain	down
	BnaA05g23450D	AT3G16250.1	Photosynthetic electron transport chain	down
	BnaC01g28660D	AT1G60600.2	Photosynthetic electron transport chain	down
	BnaC06g26170D	AT1G68010.2	Photosynthetic electron transport chain	down
	BnaCnng40790D	AT5G23060.1	Photosynthetic electron transport chain	down
	BnaC01g12690D	AT4G21280.2	Oxygen evolving enhancer protein 3	down
	BnaC01g18940D	AT4G26710.1	ATP synthase subunit H	down
	BnaC07g49690D	AT5G23240.1	Ferredoxin I	up
	BnaA03g39520D	AT5G23240.1	Ferredoxin I	up
	BnaA06g06760D	AT1G10960.1	Ferredoxin 2	down
	BnaA03g22350D	AT2G27510.1	Ferredoxin-3	up
	BnaC07g22910D	AT3G25770.1	Chloroplast thylakoid membrane	up
	BnaC01g22830D	AT4G14690.1	Chloroplast thylakoid membrane	up
	BnaA02g23180D	AT5G42650.1	Chloroplast thylakoid membrane	up
**Calvin cycle**	BnaA07g14420D	AT5G38430.1	Ribulose-1,5-bisphosphate carboxylase small subunit	down
	BnaA05g05840D	AT2G39730.1	Ribulose bisphosphate carboxylase/oxygenase activase	down
	BnaA03g18710D	AT2G39730.1	Ribulose bisphosphate carboxylase/oxygenase activase	down
	BnaC04g05700D	AT2G39730.1	Ribulose bisphosphate carboxylase/oxygenase activase	down
	BnaC03g22220D	AT2G39730.1	Ribulose bisphosphate carboxylase/oxygenase activase	down
	BnaA07g14420D	AT5G38430.1	Ribulose bisphosphate carboxylase/oxygenase activase	down
	BnaA05g33200D	AT3G04120.1	Glyceraldehyde 3-phosphate dehydrogenase, C-terminal domain	up
	BnaC05g47450D	AT1G13440.1	Glyceraldehyde 3-phosphate dehydrogenase, C-terminal domain	up
	BnaA02g28150D	AT1G12900.1	Glyceraldehyde 3-phosphate dehydrogenase, C-terminal domain	down
	BnaC01g40210D	AT3G04120.1	Glyceraldehyde 3-phosphate dehydrogenase, C-terminal domain	up
	BnaC07g13360D	AT1G23040.1	Glyceraldehyde 3-phosphate dehydrogenase, C-terminal domain	up
	BnaA07g16660D	AT3G55800.1	Fructose-1-6-bisphosphatase	down
	BnaC06g42620D	AT3G55800.1	Fructose-1-6-bisphosphatase	down
	BnaC08g35820D	AT2G21330.1	Fructose-bisphosphate aldolase class-I	down
	BnaA03g33270D	AT3G14200.1	DnaJ domain	down
	BnaAnng20610D	AT1G56300.1	DnaJ domain	up
	Bna C09g09610D	AT2G17880.1	DnaJ domain	down
	BnaC01g27270D	AT1G56300.1	DnaJ domain	up
	BnaCnng20870D	AT4G13830.2	DnaJ domain	down
	BnaC03g02360D	AT5G06110.1	DnaJ domain	up
	BnaCnng44960D	AT4G13830.2	DnaJ domain	down
	BnaA01g29880D	AT3G13310.1	DnaJ domain	up
	BnaA02g15280D	AT1G71000.1	DnaJ domain	up
	BnaAnng08930D	AT4G13830.2	DnaJ domain	down
	BnaC06g19600D	AT1G80920.1	DnaJ domain	down
	BnaA07g20150D	AT1G80920.1	DnaJ domain	down
	BnaA04g06570D	AT4G13830.2	DnaJ domain	down
	BnaC06g40110D	AT1G80920.1	DnaJ domain	down
	BnaA05g22410D	AT3G17830.1	DnaJ domain	up
	BnaA09g09350D	AT2G17880.1	DnaJ domain	down
	BnaC08g35620D	AT2G21510.1	DnaJ domain	up
	BnaC03g61170D	AT4G37910.1	Hsp70 protein	up
	BnaA02g21330D	AT4G12400.2	Hsp70 protein	down
	BnaA03g59360D		Hsp70 protein	up
	BnaA08g15870D	AT4G37910.1	Hsp70 protein	up
	BnaC09g13120D	AT1G12270.1	Hsp70 protein	up
	BnaA06g00870D	AT1G79920.1	Hsp70 protein	up
	BnaA10g27060D		Hsp70 protein	up
	BnaCnng03470D	AT3G12580.1	Hsp70 protein	up
	BnaA09g48560D	AT1G09080.1	Hsp70 protein	up
	BnaA09g12780D	AT1G62740.1	Hsp70 protein	up

### Protein–protein interaction networks in LT stress response

Protein interaction networks regulate various cellular functions, such as signal transduction, metabolic pathways, organ formation, cell cycle regulation, and plant defense (Bray, 2006; Zhang et al., 2010). The protein–protein interactions of the sugar metabolism, hormone, antioxidant activity, transcription factors, and photosynthesis-related genes revealed that the protein domains physically interact through a complex network. Specifically, the SnRK2 family contains key regulators of cellular osmotic stress and ABA responses in plants. Here the SNF1-related protein kinase 2 (SnRK2), ABI, AOS, JAZ, and HSP families shared a maximum number of interaction lines (**Figure 6**). CYP707A1 encodes abscisic acid 8’-hydroxylases, which is indispensable for seed dormancy and germination in *Arabidopsis* (Okamoto et al., 2006). Similarly, ABI5 plays a significant role in the regulation of seed germination, seedling growth, development, and abiotic stresses (Zinsmeister et al., 2016). In the ABA signal pathway, the ABI5 protein directly interacts with CYP707A1, ABI2, PP2C phosphatases, PYR/PYL/RCAR receptors, and SnRK2 kinases. The interaction of these proteins suggested that the expression of these genes is tightly associated with LT stress response. Based on these findings, ABI5 is a hub protein under LT response in *B. napus* which directly or indirectly interacts with antioxidants, TFs, ABA signaling, and regulation of LT-stress-related genes along with various other proteins. In *Arabidopsis*, alginate oligosaccharide (AOS) induced resistance to the pathogen by a salicylic-acid-mediated signaling pathway (Zhang et al., 2019a). Jasmonate and related signaling compounds have a significant role in plant immunity and development. WRKY1 negatively regulates the ABA signal pathway through JAZ1 and ABI1 in drought response (Luo et al., 2020). Moreover, AOS functions in fine-tuning JA formation and improving LT stress tolerance (Pi et al., 2009; Liu et al., 2017b). In this study, the JAZ protein directly interacts with WRKY, AOS, and other essential genes. These results showed that a complex interactive network at the protein level was activated in response to LT stress response in *B. napus*. Within the network, some proteins act as a hub to activate or repress the function of other related proteins, which helped to cope with the LT stress in *B. napus*.

## Conclusion

**Figure 9:**
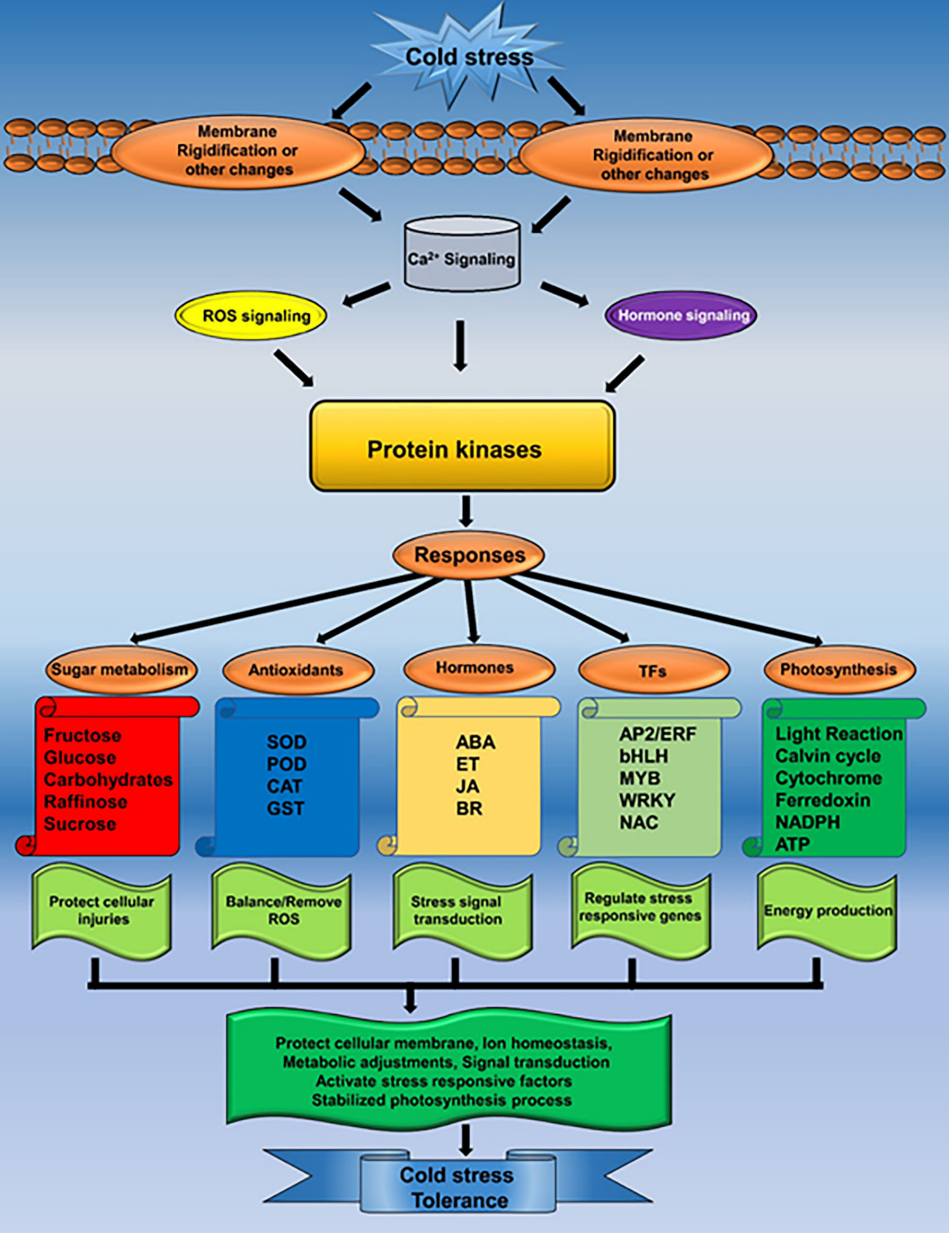
Mechanism of LT stress tolerance in rapeseed seedlings.

*B. napus* is the major oil crop with the largest planting area in China. However, its productivity and cultivation area are severely affected by LT stress. In the current study, the transcriptomic study helped to identify various LT-stress-responsive pathways and molecular events. A set of potential DEGs that respond to LT stress was discovered. Different expressions of the key systems indicated that they might play vital roles in response to LT stress in *B. napus.* It is noticeable that the *B. napus* response to LT stress is multiplex, not only regulating the genetic architecture but also activating the antioxidant and osmoprotectant system to improve the LT stress tolerance (**Figure 9**). A deep understanding of these molecular events will provide new avenues for LT stress tolerance improvement in *B. napus*.

## Data availability statement

The data presented in the study are deposited in the National Center for Biotechnology 136 Information (NCBI) repository, accession number PRJNA596550.

## Author contributions

MH and YL conceived the idea and wrote the manuscript. MH, DL, and ZL performed the experiments. DL and XD helped in the literature searches and data analysis. YC, XZ, GL, and YL supervised the work, reviewed and edited the manuscript, and managed funding resources. All authors contributed to the article and approved the submitted version.

## Funding

This work was supported by Key Research Program & Technology Innovation Program of the Chinese Academy of Agricultural Sciences (CAAS-ZDRW202109 and CAAS-ZDRW202105), the National Nature Science Foundation of China (32072131), the Key Research and Development projects in Hubei Province (2022BBA0038), and the Science and Technology Innovation Project of the Chinese Academy of Agricultural Sciences (CAAS), China, and Fundamental Research Funds for the Central Nonprofit Scientific Institution (OCRI, CAAS, 1610172018010).

## Acknowledgments

MH is thankful to CSC for providing a Ph.D. scholarship and to OCRI-CAAS for research environment. MH is thankful to GYL for article language improvement and providing APC. We would also like to thank all the members of the Key Lab of Biology and Genetic Improvement of Oil Crops, OCRI-CAAS, Wuhan, China, for their support throughout the study.

## Conflict of interest

The authors declare that the research was conducted in the absence of any commercial or financial relationships that could be construed as a potential conflict of interest.

## Publisher’s note

All claims expressed in this article are solely those of the authors and do not necessarily represent those of their affiliated organizations, or those of the publisher, the editors and the reviewers. Any product that may be evaluated in this article, or claim that may be made by its manufacturer, is not guaranteed or endorsed by the publisher.

## References

[B1] Almadanim M. C. Alexandre B. M. Rosa M. T. G. Sapeta H. Leitao A. E. Ramalho J. C.. (2017). Rice calcium-dependent protein kinase OsCPK17 targets plasma membrane intrinsic protein and sucrose-phosphate synthase and is required for a proper cold stress response. Plant Cell Environ. 40, 1197–1213. doi: 10.1111/pce.12916 28102545

[B2] An J. P. Li R. Qu F. J. You C. X. Wang X. F. Hao Y. J. (2018). An apple NAC transcription factor negatively regulates cold tolerance *via* CBF-dependent pathway. J. Plant Physiol. 221, 74–80. doi: 10.1016/j.jplph.2017.12.009 29253732

[B3] An J. P. Wang X. F. Zhang X. W. Xu H. F. Bi S. Q. You C. X.. (2020). An apple MYB transcription factor regulates cold tolerance and anthocyanin accumulation and undergoes MIEL1-mediated degradation. Plant Biotech. J. 18, 337–353. doi: 10.1111/pbi.13201 PMC6953192 31250952

[B4] An F. Zhao Q. Ji Y. Li W. Jiang Z. Yu X.. (2010). Ethylene-induced stabilization of ETHYLENE INSENSITIVE3 and EIN3-LIKE1 is mediated by proteasomal degradation of EIN3 binding f-box 1 and 2 that requires EIN2 in arabidopsis. Plant Cell. 22, 2384–2401. doi: 10.1105/tpc.110.076588 20647342 PMC2929093

[B5] Arfan M. Zhang D. W. Zou L. J. Luo S. S. Tan W. R. Zhu T.. (2019). Hydrogen peroxide and nitric oxide crosstalk mediates brassinosteroids induced cold stress tolerance in medicago truncatula. Int. J. Mol. Sci. 20, 1–15. doi: 10.3390/ijms20010144 PMC6337477 30609774

[B6] Armbruster U. Correa Galvis V. Kunz H. H. Strand D. D. (2017). The regulation of the chloroplast proton motive force plays a key role for photosynthesis in fluctuating light. Curr. Opin. Plant Biol. 37, 56–62. doi: 10.1016/j.pbi.2017.03.012 28426975

[B7] Bari R. Jones J. D. (2009). Role of plant hormones in plant defence responses. Plant Mol. Biol. 69, 473–488. doi: 10.1007/s11103-008-9435-0 19083153

[B8] Baud S. Vaultier M. N. Rochat C. (2004). Structure and expression profile of the sucrose synthase multigene family in arabidopsis. J. Exp. Bot. 55, 397–409. doi: 10.1093/jxb/erh047 14739263

[B9] Benjamini Y. Hochberg Y. (1995). Controlling the false discovery rate: a practical and powerful approach to multiple testing. J. R. Stat. Soc Ser. 57, 289–300. doi: 10.1111/j.2517-6161.1995.tb02031.x

[B10] Bray S. J. (2006). Notch signalling: a simple pathway becomes complex. Nat. Rev. Mol. Cell Biol. 7, 678–689. doi: 10.1038/nrm2009 16921404

[B11] Caverzan A. Passaia G. Rosa S. B. Ribeiro C. W. Lazzarotto F. Margis-Pinheiro M. (2012). Plant responses to stresses: Role of ascorbate peroxidase in the antioxidant protection. Genet. Mol. Biol. 35, 1011–1020. doi: 10.1590/S1415-47572012000600016 23412747 PMC3571416

[B12] Chalhoub B. Denoeud F. Liu S. Parkin I. A. Tang H. Wang X.. (2014). Plant genetics. early allopolyploid evolution in the post-neolithic brassica napus oilseed genome. Science 345, 950–953. doi: 10.1126/science.1253435 25146293

[B13] Ciereszko I. Johansson H. Kleczkowski L. A. (2001). Sucrose and light regulation of a cold-inducible UDP-glucose pyrophosphorylase gene *via* a hexokinase-independent and abscisic acid-insensitive pathway in arabidopsis. Biochem. J. 354, 67–72. doi: 10.1042/bj3540067 11171080 PMC1221629

[B14] Conesa A. Götz S. (2008). Blast2GO: A comprehensive suite for functional analysis in plant genomics. Inter J. Plant Genomics 2008, 619832. doi: 10.1155/2008/619832 PMC2375974 18483572

[B15] Cong R. H. Zhang Z. Lu J. W. (2019). Climate impacts on yield of winter oilseed rape in different growth regions of the Yangtze river basin. Chin. J. Oil Crop Science. 41, 894–903. doi: 10.19802/j.issn.1007-9084.2019046

[B16] Deng S. Ma J. Zhang L. Chen F. Sang Z. Jia Z.. (2019). *De novo* transcriptome sequencing and gene expression profiling of magnolia wufengensis in response to cold stress. BMC Plant Biol. 19, 321. doi: 10.1186/s12870-019-1933-5 31319815 PMC6637634

[B17] Ding Y. Shi Y. Yang S. (2019). Advances and challenges in uncovering cold tolerance regulatory mechanisms in plants. New Phyto. 222, 1690–1704. doi: 10.1111/nph.15696 30664232

[B18] Du C. Hu K. Xian S. Liu C. Fan J. Tu J.. (2016). Dynamic transcriptome analysis reveals AP2/ERF transcription factors responsible for cold stress in rapeseed (Brassica napus l.). Mol. Genet. Genomics 291, 1053–1067. doi: 10.1007/s00438-015-1161-0 26728151

[B19] Du X. Z. Jin Z. P. Liu D. M. Yang G. D. Pei Y. X. (2017). Hydrogen sulfide alleviates the cold stress through MPK4 in arabidopsis thaliana. Plant Physiol. Bioch. 120, 112–119. doi: 10.1016/j.plaphy.2017.09.028 29024849

[B20] Edel K. H. Kudla J. (2016). Integration of calcium and ABA signaling. Curr. Opin. Plant Biol. 33, 83–91. doi: 10.1016/j.pbi.2016.06.010 27366827

[B21] Eveland A. L. Jackson D. P. (2012). Sugars, signalling, and plant development. J. Exp. Bot. 63, 3367–3377. doi: 10.1093/jxb/err379 22140246

[B22] Filek M. Rudolphi-Skorska E. Sieprawska A. Kvasnica M. Janeczko A. (2017). Regulation of the membrane structure by brassinosteroids and progesterone in winter wheat seedlings exposed to low temperature. Steroids 128, 37–45. doi: 10.1016/j.steroids.2017.10.002 29030144

[B23] Fugate K. K. Eide J. D. Martins D. N. Grusak M. A. Deckard E. L. Finger F. L. (2019). Colocalization of sucrose synthase expression and sucrose storage in the sugarbeet taproot indicates a potential role for sucrose catabolism in sucrose accumulation. J. Plant Physiol. 240, 153016. doi: 10.1016/j.jplph.2019.153016 31400718

[B24] Gilmour S. J. Sebolt A. M. Salazar M. P. Everard J. D. Thomashow M. F. (2000). Overexpression of the arabidopsis CBF3 transcriptional activator mimics multiple biochemical changes associated with cold acclimation. Plant Physiol. 124, 1854–1865. doi: 10.1104/pp.124.4.1854 11115899 PMC59880

[B25] Guan S. Xu Q. Ma D. Zhang W. Xu Z. Zhao M.. (2019). Transcriptomics profiling in response to cold stress in cultivated rice and weedy rice. Gene 685, 96–105. doi: 10.1016/j.gene.2018.10.066 30389557

[B26] Gu H. Lu M. Zhang Z. Xu J. Cao W. Miao M. (2018). Metabolic process of raffinose family oligosaccharides during cold stress and recovery in cucumber leaves. J. Plant Physiol. 224-225, 112–120. doi: 10.1016/j.jplph.2018.03.012 29617631

[B27] Gusta L. V. Trischuk R. Weiser C. J. (2005). Plant Cold Acclimation: The Role of Abscisic Acid. Journal of Plant Growth Regulation. 24:308–318.

[B28] Hang R. Wang Z. Deng X. Liu C. Yan B. Yang C.. (2018). Ribosomal RNA biogenesis and its response to chilling stress in oryza sativa. Plant Physiol. 177, 381–397. doi: 10.1104/pp.17.01714 29555785 PMC5933117

[B29] Hauser F. Waadt R. Schroeder J. I. (2011). Evolution of abscisic acid synthesis and signaling mechanisms. Curr. Biol. 21, R346–R355. doi: 10.1016/j.cub.2011.03.015 21549957 PMC3119208

[B30] Heidarvand L. Maali A. R. (2010). What happens in plant molecular responses to cold stress? Acta Physiol. Plant 32, 419–431. doi: 10.1007/s11738-009-0451-8

[B31] Hou X. M. Zhang H. F. Liu S. Y. Wang X. K. Zhang Y. M. Meng Y. C.. (2020). The NAC transcription factor CaNAC064 is a regulator of cold stress tolerance in peppers. Plant Sci. 291, 110346. doi: 10.1016/j.plantsci.2019.110346 31928677

[B32] Huang Y. Hussain M. A. Luo D. Xu H. Zeng C. Havlickova L.. (2020). A brassica napus reductase gene dissected by associative transcriptomics enhances plant adaption to freezing stress. Front. Plant Sci. 11, 971. doi: 10.3389/fpls.2020.00971 32676095 PMC7333310

[B33] Huang X. Shi H. Hu Z. Liu A. Amombo E. Chen L.. (2017). ABA is involved in regulation of cold stress response in bermudagrass. Front. Plant Sci. 8, 1613. doi: 10.3389/fpls.2017.01613 29081782 PMC5645512

[B34] Huang Z. Zhao N. Qin M. Xu A. (2018). Mapping of quantitative trait loci related to cold resistance in brassica napus l. J. Plant Physiol. 231, 147–154. doi: 10.1016/j.jplph.2018.09.012 30268695

[B35] Hu Z. Fan J. Chen K. Amombo E. Chen L. Fu J. (2016). Effects of ethylene on photosystem II and antioxidant enzyme activity in Bermuda grass under low temperature. Photosynth Res. 128, 59–72. doi: 10.1007/s11120-015-0199-5 26497139

[B36] Hurry V. Strand A. Furbank R. Stitt M. (2000). The role of inorganic phosphate in the development of freezing tolerance and the acclimatization of photosynthesis to low temperature is revealed by the pho mutants of arabidopsis thaliana. Plant J. 24, 383–396. doi: 10.1046/j.1365-313x.2000.00888.x 11069711

[B37] Hwang I. Manoharan R. K. Kang J. G. Chung M. Y. Kim Y. W. Nou I. S. (2016). Genome-wide identification and characterization of bZIP transcription factors in brassica oleracea under cold stress. BioMed. Res. Int. 2016, 4376598. doi: 10.1155/2016/4376598 27314020 PMC4893578

[B38] Ishitani M. Xiong L. Stevenson B. Zhu J. K. (1997). Genetic analysis of osmotic and cold stress signal transduction in Arabidopsis: interactions and convergence of abscisic acid-dependent and abscisic acid-independent pathways. Plant Cell. 9, 1935–1949. 9401119 10.1105/tpc.9.11.1935 PMC157048

[B39] Jaglo K. R. Kleff S. Amundsen K. L. Zhang X. Haake V. Zhang J. Z.. (2001). Components of the arabidopsis c-repeat/dehydration-responsive element binding factor cold-response pathway are conserved in brassica napus and other plant species. Plant Physiol. 127, 910–917. doi: 10.1104/pp.010548 11706173 PMC129262

[B40] Jaikumar N. S. Snapp S. S. Sharkey T. D. (2016). Older thinopyrum intermedium (Poaceae) plants exhibit superior photosynthetic tolerance to cold stress and greater increases in two photosynthetic enzymes under freezing stress compared with young plants. J. Exp. Bot. 67, 4743–4753. doi: 10.1093/jxb/erw253 27401911 PMC4973744

[B41] Janmohammadi M. Zolla L. Rinalducci S. (2015). Low temperature tolerance in plants: changes at the protein level. Phytochemistry 117, 76–89. doi: 10.1016/j.phytochem.2015.06.003 26068669

[B42] Ju C. Yoon G. M. Shemansky J. M. Lin D. Y. Ying Z. I. Chang J.. (2012). CTR1 phosphorylates the central regulator EIN2 to control ethylene hormone signaling from the ER membrane to the nucleus in arabidopsis. Proc. Natl. Acad. Sci. U S A. 109, 19486–19491. doi: 10.1073/pnas.1214848109 23132950 PMC3511113

[B43] Kaplan F. Kopka J. Sung D. Y. Zhao W. Popp M. Porat R.. (2007). Transcript and metabolite profiling during cold acclimation of arabidopsis reveals an intricate relationship of cold-regulated gene expression with modifications in metabolite content. Plant J. 50, 967–981. doi: 10.1111/j.1365-313X.2007.03100.x 17461790

[B44] Katsir L. Schilmiller A. L. Staswick P. E. He S. Y. Howe G. A. (2008). COI1 is a critical component of a receptor for jasmonate and the bacterial virulence factor coronatine. Proc Natl Acad Sci U S A. 105, 7100–7105. 18458331 10.1073/pnas.0802332105 PMC2383947

[B45] Ke L. Lei W. Yang W. Wang J. Gao J. Cheng J.. (2020). Genome-wide identification of cold responsive transcription factors in *Brassica napus* l. BMC Plant Biol. 20, 62. doi: 10.1186/s12870-020-2253-5 32028890 PMC7006134

[B46] Kong F. Deng Y. Zhou B. Wang G. Wang Y. Meng Q. (2014). A chloroplast-targeted DnaJ protein contributes to maintenance of photosystem II under chilling stress. J. Exp. Bot. 65, 143–158. doi: 10.1093/jxb/ert357 24227338 PMC3883286

[B47] Kroll J. S. Langford P. R. Wilks K. E. Keil A. D. (1995). Bacterial [Cu,Zn]-superoxide dismutase: phylogenetically distinct from the eukaryotic enzyme, and not so rare after all! Microbiology 141, 2271–2279. doi: 10.1099/13500872-141-9-2271 7496539

[B48] Ku Y. S. Sintaha M. Cheung M. Y. Lam H. M. (2018). Plant hormone signaling crosstalks between biotic and abiotic stress responses. Int. J. Mol. Sci. 19, 1–35. doi: 10.3390/ijms19103206 PMC6214094 30336563

[B49] Liao X. Li M. Liu B. Yan M. Yu X. Zi H.. (2018). Interlinked regulatory loops of ABA catabolism and biosynthesis coordinate fruit growth and ripening in woodland strawberry. Proc. Natl. Acad. Sci. U S A. 115, E11542–E11550. doi: 10.1073/pnas.1812575115 30455308 PMC6298082

[B50] Li H. Dong Y. Chang J. He J. Chen H. Liu Q.. (2016a). High-throughput MicroRNA and mRNA sequencing reveals that MicroRNAs may be involved in melatonin-mediated cold tolerance in citrullus lanatus l. Front. Plant Sci. 7, 1231. doi: 10.3389/fpls.2016.01231 27574526 PMC4983558

[B51] Li W. Huai X. Li P. Raza A. Mubarik M. S. Habib M.. (2021). Genome-wide characterization of glutathione peroxidase (GPX) gene family in rapeseed (Brassica napus l.) revealed their role in multiple abiotic stress response and hormone signaling. Antioxidants (Basel). 10. doi: 10.3390/antiox10091481 PMC8472808 34573113

[B52] Li Z. Hu G. Liu X. Zhou Y. Li Y. Zhang X.. (2016b). Transcriptome sequencing identified genes and gene ontologies associated with early freezing tolerance in maize. Front. Plant Sci. 7, 1477. doi: 10.3389/fpls.2016.01477 27774095 PMC5054024

[B53] Li C. Jia H. Chai Y. Shen Y. (2011). Abscisic acid perception and signaling transduction in strawberry: a model for non-climacteric fruit ripening. Plant Signal. Behav. 6, 1950–1953. doi: 10.4161/psb.6.12.18024 22095148 PMC3337185

[B54] Li Y. Li L. Ding W. Li H. Shi T. Yang X.. (2020). Genome-wide identification of osmanthus fragrans bHLH transcription factors and their expression analysis in response to abiotic stress. Environ. Exp. Botany. 172, 103990. doi: 10.1016/j.envexpbot.2020.103990

[B55] Li N. N. Qian W. J. Wang L. Cao H. L. Hao X. Y. Yang Y. J.. (2017b). Isolation and expression features of hexose kinase genes under various abiotic stresses in the tea plant (Camellia sinensis). J. Plant Physiol. 209, 95–104. doi: 10.1016/j.jplph.2016.11.007 28013175

[B56] Liu W. H. Cheng C. Z. Chen F. L. Ni S. S. Lin Y. L. Lai Z. X. (2018a). High-throughput sequencing of small RNAs revealed the diversified cold-responsive pathways during cold stress in the wild banana (Musa itinerans). BMC Plant Biol. 18, 1–26. doi: 10.1186/s12870-018-1483-2 30486778 PMC6263057

[B57] Liu T. Fang H. Liu J. Reid S. Hou J. Zhou T.. (2017a). Cytosolic glyceraldehyde-3-phosphate dehydrogenases play crucial roles in controlling cold-induced sweetening and apical dominance of potato (Solanum tuberosum l.) tubers. Plant Cell Environ. 40, 3043–3054. doi: 10.1111/pce.13073 28940493

[B58] Liu C. Feng Z. C. Xiao T. H. Ma X. M. Zhou G. S. Huang F. H.. (2019). Development, potential and adaption of Chinese rapeseed industry. Chin. J. Oil Crop Science. 41, 485–489. doi: 10.7505/j.issn.1007-9084.2019.04.001

[B59] Liu X. Lan J. Huang Y. Cao P. Zhou C. Ren Y.. (2018b). WSL5, a pentatricopeptide repeat protein, is essential for chloroplast biogenesis in rice under cold stress. J. Exp. Bot. 69, 3949–3961. doi: 10.1093/jxb/ery214 29893948 PMC6054151

[B60] Liu W. Wang H. Chen Y. Zhu S. Chen M. Lan X.. (2017b). Cold stress improves the production of artemisinin depending on the increase in endogenous jasmonate. Biotechnol. Appl. Biochem. 64, 305–314. doi: 10.1002/bab.1493 26988377

[B61] Liu Y. Zhang L. Chen L. Ma H. Ruan Y. Xu T.. (2016). Molecular cloning and expression of an encoding galactinol synthase gene (AnGolS1) in seedling of ammopiptanthus nanus. Sci. Rep. 6, 36113. doi: 10.1038/srep36113 27786294 PMC5081558

[B62] Li H. Ye K. Shi Y. Cheng J. Zhang X. Yang S. (2017a). BZR1 positively regulates freezing tolerance *via* CBF-dependent and CBF-independent pathways in arabidopsis. Mol. Plant 10, 545–559. doi: 10.1016/j.molp.2017.01.004 28089951

[B63] Li P. Zheng T. Li L. Zhuo X. Jiang L. Wang J.. (2019). Identification and comparative analysis of the CIPK gene family and characterization of the cold stress response in the woody plant prunus mume. PeerJ. 7, e6847. doi: 10.7717/peerj.6847 31106064 PMC6499057

[B64] Lu S. Fadlalla T. Tang S. Li L. Ali U. Li Q.. (2019). Genome-wide analysis of phospholipase d gene family and profiling of phospholipids under abiotic stresses in brassica napus. Plant Cell Physiol. 60, 1556–1566. doi: 10.1093/pcp/pcz071 31073607

[B65] Luo X. Li C. He X. Zhang X. Zhu L. (2020). ABA signaling is negatively regulated by GbWRKY1 through JAZ1 and ABI1 to affect salt and drought tolerance. Plant Cell Rep. 39, 181–194. doi: 10.1007/s00299-019-02480-4 31713664

[B66] Luo T. Xian M. Zhang C. Zhang C. Hu L. Xu Z. (2019). Associating transcriptional regulation for rapid germination of rapeseed (*Brassica napus* l.) under low temperature stress through weighted gene co-expression network analysis. Sci. Rep. 9, 55. doi: 10.1038/s41598-018-37099-0 30635606 PMC6329770

[B67] Lv Y. Yang M. Hu D. Yang Z. Ma S. Li X.. (2017). The OsMYB30 transcription factor suppresses cold tolerance by interacting with a JAZ protein and suppressing β-amylase expression. Plant Physiol. 173, 1475–1491. doi: 10.1104/pp.16.01725 28062835 PMC5291022

[B68] Ma X. Chen C. Yang M. Dong X. Lv W. Meng Q. (2018). Cold-regulated protein (SlCOR413IM1) confers chilling stress tolerance in tomato plants. Plant Physiol. Biochem. 124, 29–39. doi: 10.1016/j.plaphy.2018.01.003 29331923

[B69] Ma L. Coulter J. A. Liu L. Zhao Y. Chang Y. Pu Y.. (2019). Transcriptome analysis reveals key cold-Stress-Responsive genes in winter rapeseed (*Brassica rapa* L.). Int. J. Mol. Sci. 20, 1–19. doi: 10.3390/ijms20051071 PMC6429191 30832221

[B70] Mao X. Z. Cai T. Olyarchuk J. G. Wei L. P. (2005). Automated genome annotation and pathway identification using the KEGG orthology (KO) as a controlled vocabulary. Bioinformatics 21, 3787–3793. doi: 10.1093/bioinformatics/bti430 15817693

[B71] Maryan K. E. Lahiji H. S. Farrokhi N. Komeleh H. H. (2019). Analysis of brassica napus dehydrins and their Co-expression regulatory networks in relation to cold stress. Gene Expr Patterns. 31, 7–17. doi: 10.1016/j.gep.2018.10.002 30408599

[B72] Miller G. Suzuki N. Ciftci-Yilmaz S. Mittler R. (2010). Reactive oxygen species homeostasis and signalling during drought and salinity stresses. Plant Cell Environ. 33, 453–467. doi: 10.1111/j.1365-3040.2009.02041.x 19712065

[B73] Mizoi J. Shinozaki K. Yamaguchi-Shinozaki K. (2012). AP2/ERF family transcription factors in plant abiotic stress responses. Biochim. Biophys. Acta 1819, 86–96. doi: 10.1016/j.bbagrm.2011.08.004 21867785

[B74] Murata N. Nishiyama Y. (2018). ATP is a driving force in the repair of photosystem II during photoinhibition. Plant Cell Environ. 41, 285–299. doi: 10.1111/pce.13108 29210214

[B75] Nishiyama Y. Murata N. (2014). Revised scheme for the mechanism of photoinhibition and its application to enhance the abiotic stress tolerance of the photosynthetic machinery. Appl. Microbiol. Biotechnol. 98, 8777–8796. doi: 10.1007/s00253-014-6020-0 25139449

[B76] Niu X. Lu H. Fan Y. Wang W. Yuan Y. Hawkins M.. (2022). Manipulation of the transcription factor SlNAC1 for improved tolerance to abiotic stress in tomato. Plant Cell Environ. 1–14. doi: 10.1111/pce.14437 36128662

[B77] Nowicka B. Kruk J. (2018). [Genetic engineering as a method for the improvement of photosynthesis]. Postepy Biochem. 64, 13–20. doi: 10.18388/pb.2018_100 30652833

[B78] Oelze M. L. Kandlbinder A. D. K. J. (2008). Redox regulation and over reduction control in the photosynthesizing cell: complexity in redox regulatory networks. BBA Gen. Subj. 1780, 1261–1272. doi: 10.1016/j.bbagen.2008.03.015 18439433

[B79] Okamoto M. Kuwahara A. Seo M. Kushiro T. Asami T. Hirai N.. (2006). CYP707A1 and CYP707A2, which encode abscisic acid 8'-hydroxylases, are indispensable for proper control of seed dormancy and germination in arabidopsis. Plant Physiol. 141, 97–107. doi: 10.1104/pp.106.079475 16543410 PMC1459320

[B80] Paredes M. Quiles M. J. (2015). The effects of cold stress on photosynthesis in hibiscus plants. PloS One 10, e0137472. doi: 10.1371/journal.pone.0137472 26360248 PMC4567064

[B81] Pi Y. Jiang K. Cao Y. Wang Q. Huang Z. Li L.. (2009). Allene oxide cyclase from camptotheca acuminata improves tolerance against low temperature and salt stress in tobacco and bacteria. Mol. Biotechnol. 41, 115–122. doi: 10.1007/s12033-008-9106-z 18850307

[B82] Pradhan S. K. Pandit E. Nayak D. K. Behera L. Mohapatra T. (2019). Genes, pathways and transcription factors involved in seedling stage chilling stress tolerance in indica rice through RNA-seq analysis. BMC Plant Biol. 19, 352. doi: 10.1186/s12870-019-1922-8 31412781 PMC6694648

[B83] Pu Y. Liu L. Wu J. Zhao Y. Bai J. Ma L.. (2019). Transcriptome profile analysis of winter wapeseed (*Brassica napus* L.) in response to freezing stress, reveal potentially connected events to freezing stress. Int. J. Mol. Sci. 20, 1–24. doi: 10.3390/ijms20112771 PMC6600501 31195741

[B84] Qi J. Song C. P. Wang B. Zhou J. Kangasjärvi J. Zhu J. K.. (2018). Reactive oxygen species signaling and stomatal movement in plant responses to drought stress and pathogen attack. J. Integr. Plant Biol. 60, 805–826. doi: 10.1111/jipb.12654 29660240

[B85] Raza A. Su W. Hussain M. A. Mehmood S. S. Zhang X. Cheng Y.. (2021). Integrated analysis of metabolome and transcriptome reveals insights for cold tolerance in rapeseed (Brassica napus l.). Front. Plant Sci. 12, 721681. doi: 10.3389/fpls.2021.721681 34691103 PMC8532563

[B86] Salvi P. Saxena S. C. Petla B. P. Kamble N. U. Kaur H. Verma P.. (2016). Differentially expressed galactinol synthase(s) in chickpea are implicated in seed vigor and longevity by limiting the age induced ROS accumulation. Sci. Rep. 6, 35088. doi: 10.1038/srep35088 27725707 PMC5057127

[B87] Schieber M. Chandel N. S. (2014). ROS function in redox signaling and oxidative stress. Curr. Biol. 24, R453–R462. doi: 10.1016/j.cub.2014.03.034 24845678 PMC4055301

[B88] Seiler C. Harshavardhan V. T. Rajesh K. Reddy P. S. Strickert M. Rolletschek H.. (2011). ABA biosynthesis and degradation contributing to ABA homeostasis during barley seed development under control and terminal drought-stress conditions. J. Exp. Bot. 62, 2615–2632. doi: 10.1093/jxb/erq446 21289079

[B89] Sharma T. R. Devanna B. N. Kiran K. Singh P. K. Arora K. Jain P.. (2018). Status and prospects of next generation sequencing technologies in crop plants. Curr. Issues Mol. Biol. 27, 1–36. doi: 10.21775/cimb.027.001 28885172

[B90] Sharma A. Shahzad B. Kumar V. Kohli S. K. Sidhu G. P. S. Bali A. S.. (2019). Phytohormones regulate accumulation of osmolytes under abiotic stress. Biomolecules 9, 1–36. doi: 10.3390/biom9070285 PMC6680914 31319576

[B91] Shi Y. Cai Z. Li D. Lu J. Ye J. Liang Y.. (2019). Effect of freezing on photosystem II and assessment of freezing tolerance of tea cultivar. Plants 8, 1–14. doi: 10.3390/plants8100434 PMC6843692 31652528

[B92] Shi Y. Ding Y. Yang S. (2018). Molecular regulation of CBF signaling in cold acclimation. Trends Plant Sci. 23, 623–637. doi: 10.1016/j.tplants.2018.04.002 29735429

[B93] Sicher R. (2011). Carbon partitioning and the impact of starch deficiency on the initial response of arabidopsis to chilling temperatures. Plant Sci. 181, 167–176. doi: 10.1016/j.plantsci.2011.05.005 21683882

[B94] Sies H. Carsten B. Dean P. J. (2017). Oxidative stress. Annu. Rev. Biochem. 86, 715–748. doi: 10.1146/annurev-biochem-061516-045037 28441057

[B95] Sirichandra C. Gu D. Hu H. C. Davanture M. Lee S. Djaoui M.. (2009). Phosphorylation of the arabidopsis AtrbohF NADPH oxidase by OST1 protein kinase. FEBS Lett. 583, 2982–2986. doi: 10.1016/j.febslet.2009.08.033 19716822

[B96] Skubacz A. Daszkowska-Golec A. Szarejko I. (2016). The role and regulation of ABI5 (ABA-insensitive 5) in plant development, abiotic stress responses and phytohormone crosstalk. Front. Plant Sci. 7, 1884. doi: 10.3389/fpls.2016.01884 28018412 PMC5159420

[B97] Song J. Zeng L. Chen R. Wang Y. Zhou Y. (2018). In silico identification and expression analysis of superoxide dismutase (SOD) gene family in medicago truncatula. 3 Biotech. 8, 348. doi: 10.1007/s13205-018-1373-1 PMC6066495 30073133

[B98] Stein O. Granot D. (2019). An overview of sucrose synthases in plants. Front. Plant Sci. 10, 95. doi: 10.3389/fpls.2019.00095 30800137 PMC6375876

[B99] Strand A. Hurry V. Henkes S. Huner N. Gustafsson P. Gardeström P.. (1999). Acclimation of arabidopsis leaves developing at low temperatures. increasing cytoplasmic volume accompanies increased activities of enzymes in the Calvin cycle and in the sucrose-biosynthesis pathway. Plant Physiol. 119, 1387–1398. doi: 10.1104/pp.119.4.1387 10198098 PMC32024

[B100] Su C. Chen K. Ding Q. Mou Y. Yang R. Zhao M.. (2018). Proteomic analysis of the function of a novel cold-regulated multispanning transmembrane protein COR413-PM1 in arabidopsis. Int. J. Mol. Sci. 19, 1–22. doi: 10.3390/ijms19092572 PMC6165019 30158496

[B101] Sun X. Wang Y. Sui N. (2018). Transcriptional regulation of bHLH during plant response to stress. Biochem. Biophys. Res. Commun. 503, 397–401. doi: 10.1016/j.bbrc.2018.07.123 30057319

[B102] Sun H. Zhang W. Tang L. Han S. Wang X. Zhou S.. (2015). Investigation of the role of the calvin cycle and C1 metabolism during HCHO metabolism in gaseous HCHO-treated petunia under light and dark conditions using 13C-NMR. Phytochem. Anal. 26, 226–235. doi: 10.1002/pca.2556 25693735

[B103] Sun X. Zhang L. Wong D. C. J. Wang Y. Zhu Z. Xu G.. (2019). The ethylene response factor VaERF092 from amur grape regulates the transcription factor VaWRKY33, improving cold tolerance. Plant J. 99, 988–1002. doi: 10.1111/tpj.14378 31063661

[B104] Sun X. Zhao T. Gan S. Ren X. Fang L. Karungo S. K.. (2016). Ethylene positively regulates cold tolerance in grapevine by modulating the expression of ETHYLENE RESPONSE FACTOR 057. Sci. Rep. 6, 24066. doi: 10.1038/srep24066 27039848 PMC4819186

[B105] Su W. Raza A. Gao A. Jia Z. Zhang Y. Hussain M. A.. (2021). Genome-wide analysis and expression profile of superoxide dismutase (SOD) gene family in rapeseed (Brassica napus l.) under different hormones and abiotic stress conditions. Antioxidants (Basel). 10. doi: 10.3390/antiox10081182 PMC8389029 34439430

[B106] Suzuki N. Mittler R. (2006). Reactive oxygen species and temperature stresses: A delicate balance between signaling and destruction. Physiologia Plantarum. 126, 45–51. doi: 10.1111/j.0031-9317.2005.00582.x

[B107] Trapnell C. (2010). Transcript assembly and quantification by RNA-seq reveals unannotated transcripts and isoform switching during cell differentiation. Nat. Biotechnol. . 28, 511–515. doi: 10.1038/nbt.1621 20436464 PMC3146043

[B108] Tyczewska A. Gracz J. Kuczynski J. Twardowski T. (2016). Deciphering the soybean molecular stress response *via* high-throughput approaches. Acta Biochim. Pol. 63, 631–643. doi: 10.18388/abp.2016_1340 27851833

[B109] Verma V. Ravindran P. Kumar P. P. (2016). Plant hormone-mediated regulation of stress responses. BMC Plant Biol. 16, 86. doi: 10.1186/s12870-016-0771-y 27079791 PMC4831116

[B110] Wang Z. Chen Y. Fang H. D. Shi H. F. Chen K. P. Zhang Z. Y.. (2014). Selection of reference genes for quantitative reverse-transcription polymerase chain reaction normalization in brassica napus under various stress conditions. Mol. Genet. Genomics 289, 1023–1035. doi: 10.1007/s00438-014-0853-1 24770781

[B111] Wang L. B. Wang L. Zhang Z. E. Ma M. Wang R. Z. Qian M.. (2018a). Genome-wide identification and comparative analysis of the superoxide dismutase gene family in pear and their functions during fruit ripening. Postharvest Biol. Technol. 143, 68–77. doi: 10.1016/j.postharvbio.2018.04.012

[B112] Wang T. Song H. Zhang B. Lu Q. Liu Z. Zhang S.. (2018b). Genome-wide identification, characterization, and expression analysis of superoxide dismutase (SOD) genes in foxtail millet (Setaria italica l.). Biotech. 8, 486. doi: 10.1007/s13205-018-1502-x PMC6240016 30498660

[B113] Wittkopp P. J. Kalay G. (2012). Cis-regulatory elements: molecular mechanisms and evolutionary processes underlying divergence. Nat. Rev. Genet. 13, 59–69. doi: 10.1038/nrg3095 22143240

[B114] Wu J. Seng S. Sui J. Vonapartis E. Luo X. Gong B.. (2015). Gladiolus hybridus ABSCISIC ACID INSENSITIVE 5 (GhABI5) is an important transcription factor in ABA signaling that can enhance gladiolus corm dormancy and arabidopsis seed dormancy. Front. Plant Sci. 6, 960. doi: 10.3389/fpls.2015.00960 26579187 PMC4630654

[B115] Xin H. Xianchao N. Pan X. Wei L. Min Y. Yu K.. (2019). Comparative transcriptome analyses revealed conserved and novel responses to cold and freezing stress in *Brassica napus* l. G3 (Bethesda). 9, 2723–2737. doi: 10.1534/g3.119.400229 31167831 PMC6686917

[B116] Xiong H. Wang R. Jia X. Sun H. Duan R. (2022). Transcriptomic analysis of rapeseed (Brassica napus. l.) seed development in xiangride, qinghai plateau, reveals how its special eco-environment results in high yield in high-altitude areas. Front. Plant Sci. 13, 927418. doi: 10.3389/fpls.2022.927418 35982704 PMC9379305

[B117] Yang C. Lu X. Ma B. Chen S. Y. Zhang J. S. (2015). Ethylene signaling in rice and arabidopsis: conserved and diverged aspects. Mol. Plant 8, 495–505. doi: 10.1016/j.molp.2015.01.003 25732590

[B118] Yan L. Shah T. Cheng Y. Lu Y. Zhang X. K. Zou X. L. (2019). Physiological and molecular responses to cold stress in rapeseed (Brassica napus l.). J. Integr. Agr. 18, 2742–2752. doi: 10.1016/S2095-3119(18)62147-1

[B119] Young M. D. Wakefield M. J. Smyth G. K. (2010). Gene ontology analysis for RNA-seq: accounting for selection bias. Genome Biol. Evol. 11, 1–12. doi: 10.1186/gb-2010-11-2-r14 PMC2872874 20132535

[B120] Yu H. Kong X. Huang H. Wu W. Park J. Yun D. J.. (2020). STCH4/REIL2 confers cold stress tolerance in arabidopsis by promoting rRNA processing and CBF protein translation. Cell Rep. 30, 229–242. doi: 10.1016/j.celrep.2019.12.012 31914389

[B121] Yu J. Xu S. Liu X. Li T. Zhang D. Teng N.. (2022a). Starch degradation and sucrose accumulation of lily bulbs after cold storage. Int. J. Mol. Sci. 23, 1–17. doi: 10.3390/ijms23084366 PMC9029042 35457184

[B122] Yu Q. Zheng Q. Shen W. Li J. Yao W. Xu W. (2022b). Grape CIPK18 acts as a positive regulator of CBF cold signaling pathway by modulating ROS homeostasis. Environ. Exper Bot. 203, 105063. doi: 10.1016/j.envexpbot.2022.105063

[B123] Zhang J. An H. Zhang X. Xu F. Zhou B. (2022a). Transcriptomic analysis reveals potential gene regulatory networks under cold stress of loquat (Eriobotrya japonica lindl.). Front. Plant Sci. 13, 944269. doi: 10.3389/fpls.2022.944269 35937353 PMC9354853

[B124] Zhang Y. Gao P. Yuan J. S. (2010). Plant protein-protein interaction network and interactome. Curr. Genomics 11, 40–46. doi: 10.2174/138920210790218016 20808522 PMC2851115

[B125] Zhang C. Howlader P. Liu T. Sun X. Jia X. Zhao X.. (2019a). Alginate oligosaccharide (AOS) induced resistance to pst DC3000 *via* salicylic acid-mediated signaling pathway in arabidopsis thaliana. Carbohydr Polym. 225, 115221. doi: 10.1016/j.carbpol.2019.115221 31521273

[B126] Zhang Y. Launay H. Liu F. Lebrun R. Gontero B. (2018). Interaction between adenylate kinase 3 and glyceraldehyde-3-phosphate dehydrogenase from chlamydomonas reinhardtii. FEBS J. 285, 2495–2503. doi: 10.1111/febs.14494 29727516

[B127] Zhang J. Li X. M. Lin H. X. Chong K. (2019b). Crop improvement through temperature resilience. Annu. Rev. Plant Biol. 70, 753–780. doi: 10.1146/annurev-arplant-050718-100016 31035832

[B128] Zhang L. Song J. Lin R. Tang M. Shao S. Yu J.. (2022b). The SlMYB15 transcription factor targeted by sly-miR156e-3p positively regulates ABA-mediated cold tolerance in tomato. J. Exp. Bot. erac370. doi: 10.1093/jxb/erac370 36103722

[B129] Zhang W. Wang S. Yu F. Tang J. Shan X. Bao K.. (2019c). Genome-wide characterization and expression profiling of SWEET genes in cabbage (Brassica oleracea var. capitata l.) reveal their roles in chilling and clubroot disease responses. BMC Genomics 20, 93. doi: 10.1186/s12864-019-5454-2 30696401 PMC6352454

[B130] Zhang D. Ye H. Guo H. Johnson A. Zhang M. Lin H.. (2014). Transcription factor HAT1 is phosphorylated by BIN2 kinase and mediates brassinosteroid repressed gene expression in arabidopsis. Plant J. 77, 59–70. doi: 10.1111/tpj.12368 24164091

[B131] Zhao M. Liu W. Xia X. Wang T. Zhang W. H. (2014). Cold acclimation-induced freezing tolerance of medicago truncatula seedlings is negatively regulated by ethylene. Physiol. Plant 152, 115–129. doi: 10.1111/ppl.12161 24494928

[B132] Zhao J. Zhang S. Yang T. Zeng Z. Huang Z. Liu Q.. (2015). Global transcriptional profiling of a cold-tolerant rice variety under moderate cold stress reveals different cold stress response mechanisms. Physiol. Plant 154, 381–394. doi: 10.1111/ppl.12291 25263631

[B133] Zheng Y. Luo L. Wei J. Chen Q. Yang Y. Hu X.. (2018). The glutamate receptors AtGLR1.2 and AtGLR1.3 increase cold tolerance by regulating jasmonate signaling in arabidopsis thaliana. Biochem. Biophys. Res. Commun. 506, 895–900. doi: 10.1016/j.bbrc.2018.10.153 30392908

[B134] Zhuo X. Zheng T. Zhang Z. Zhang Y. Jiang L. Ahmad S.. (2018). Genome-wide analysis of the NAC transcription factor gene family reveals differential expression patterns and cold-stress responses in the woody plant prunus mume. Genes 9, 1–22. doi: 10.3390/genes9100494 PMC6209978 30322087

[B135] Zinsmeister J. Lalanne D. Terrasson E. Chatelain E. Vandecasteele C. Vu B. L.. (2016). ABI5 is a regulator of seed maturation and longevity in legumes. Plant Cell. 28, 2735–2754. doi: 10.1105/tpc.16.00470 27956585 PMC5155344

[B136] Zurbriggen M. D. Tognetti V. B. Fillat M. F. Hajirezaei M. R. Valle E. M. Carrillo N. (2008). Combating stress with flavodoxin: A promising route for crop improvement. Trends Biotechnol. 26, 531–537. doi: 10.1016/j.tibtech.2008.07.001 18706721

