# Supplementary figures and images for: Genome-wide transcriptome profiling revealed biological macromolecules respond to low temperature stress in Brassica napus L
Source: Front Plant Sci. 2022 Nov 14;13:1050995. doi: 10.3389/fpls.2022.1050995 (PMC9702069; doi:10.3389/fpls.2022.1050995)

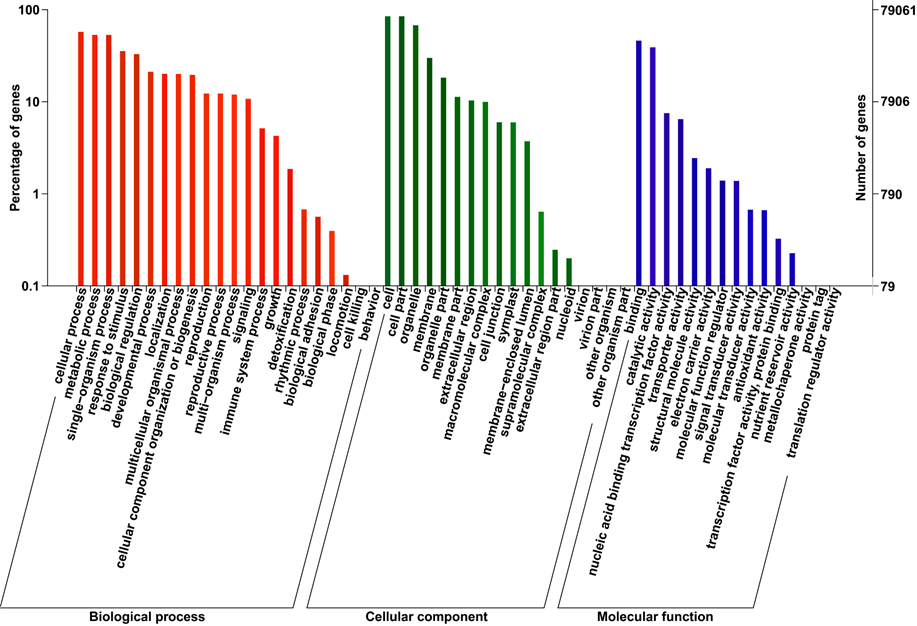

Supplement: Supplementary file 1 [file Image_1.jpeg]
